# Expression and activity of heterologous hydroxyisocaproate dehydrogenases in *Synechocystis* sp. PCC 6803 Δ*hoxYH*

**DOI:** 10.1016/j.engmic.2021.100008

**Published:** 2021-11-26

**Authors:** Valentina Jurkaš, Christoph K. Winkler, Silvan Poschenrieder, Paulo Oliveira, Catarina C. Pacheco, Eunice A. Ferreira, Florian Weissensteiner, Piera De Santis, Selin Kara, Robert Kourist, Paula Tamagnini, Wolfgang Kroutil

**Affiliations:** a Institute of Chemistry, University of Graz, NAWI Graz, Heinrichstrasse 28, 8010 Graz, Austria.; b i3S – Instituto de Investigação e Inovação em Saúde, Universidade do Porto, 4200-135 Porto, Portugal.; c IBMC – Instituto de Biologia Molecular e Celular, Universidade do Porto, 4200-135 Porto, Portugal.; d Departamento de Biologia, Faculdade de Ciências, Universidade do Porto, 4169-007 Porto, Portugal.; e ICBAS – Instituto de Ciências Biomédicas Abel Salazar, Universidade do Porto, 4050-313 Porto, Portugal.; f Aarhus University, Department of Engineering, Biological and Chemical Engineering Section, Biocatalysis and Bioprocessing Group, Gustav Wieds Vej 10, DK 8000 Aarhus, Denmark.; g Institute of Molecular Biotechnology, Graz University of Technology, 8010 Graz, Austria.; h Field of Excellence BioHealth, University of Graz, 8010 Graz, Austria.; i BioTechMed Graz, 8010 Graz, Austria.

**Keywords:** Cyanobacteria, Biocatalysis, Photobiotransformation, *Synechocystis* sp. PCC 6803, Hydroxyisocaproate dehydrogenase, Ketoacid dehydrogenase

## Abstract

Exploiting light to drive redox reactions is currently a hot topic since light is considered as an environmentally friendly source of energy. Consequently, cyanobacteria, which can use light e.g., for generating NADPH, are in the focus of research. Previously, it has been shown that various heterologous redox enzymes could be expressed in these microorganisms. Here we demonstrated the successful inducer-free expression of α-keto-acid dehydrogenases (L-HicDH and D-HicDH) from *Lactobacillus confusus* DSM 20196 and *Lactobacillus paracasei* DSM 20008 in *Synechocystis* sp. PCC 6803 Δ*hoxYH* mutant using replicative plasmids. While the L-HicDH showed poor activity limited by the amount of expressed enzyme, the D-HicDH was applied both *in vivo* and *in vitro*, transforming the selected α-keto acids to the corresponding optically pure (*R*)-α-hydroxy acids (*ee* >99%) in up to 53% and 90% conversion, respectively.

## graphical abstract

**Figure:**
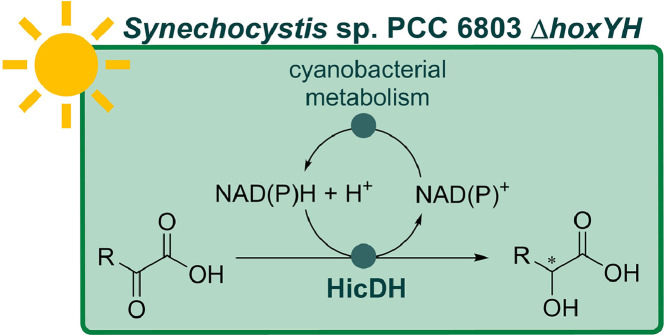

## 1 Introduction

Cyanobacteria are microorganisms that rely on oxygenic photosynthesis for growth and survival. Besides some minerals they require only sunlight and carbon dioxide as energy and carbon source, respectively. Compared to higher plants and eukaryotic microalgae, prokaryotic cyanobacteria are well-known for rapid growth and a relatively simple genetic background amenable to manipulation. Thus, the simple nutritional requirements of these organisms, combined with the autotrophic lifestyle and metabolic plasticity, as well as the availability of molecular and synthetic biology tools make them promising ‘low-cost’ solar-driven microbial cell factories (Abed et al., 2009; Carroll et al., 2018; Knoot et al., 2018; Lau et al., 2015; Santos-Merino et al., 2019; Thajuddin and Subramanian, 2005).

In recent years, the development of molecular tools enabled the engineering of new recombinant photoautotrophic strains by expressing heterologous oxidoreductive enzymes that benefit from the steady photosynthetic production of NADPH (Jodlbauer et al., 2021; Schmermund et al., 2019). This paved the way to develop new approaches for the recycling of the costly cofactors NADH and NADPH. Such phototrophic regeneration of NADPH uses only light and water as stoichiometric reagents, providing superior atom economy in comparison to traditional industrially applied approaches (Mordhorst and Andexer, 2020). Examples include an ene reductase (Assil-Companioni et al., 2020; Köninger et al., 2016), a Baeyer-Villiger monooxygenase (Böhmer et al., 2017), a carboxylic acid reductase (Yunus and Jones, 2018), the AlkBGT hydroxylation-system (Hoschek et al., 2019a), a cytochrome P450 monooxygenase (Hoschek et al., 2019b), an alcohol dehydrogenase (Sengupta et al., 2019) and imine reductases (Büchsenschütz et al., 2020). However, strategies for tightly controlled gene expression, that are widely available and routinely used in *E. coli* and other heterotrophic hosts, are still very limited for cyanobacteria (Hitchcock et al., 2020; Jodlbauer et al., 2021; Till et al., 2020). Foreign DNA instability and particularly expression of toxic enzymes can therefore pose a particular challenge (Borirak et al., 2015; Jones, 2014; Keasling, 2008; Wu et al., 2020; Yunus and Jones, 2018).

The best-studied cyanobacterial strain to date is the unicellular non-nitrogen-fixing *Synechocystis* sp. PCC 6803, widely acknowledged as the ‘green *E. coli*’ (Branco dos Santos et al., 2014). The vast amount of physiological and molecular data available, together with a relatively small genome, make *Synechocystis* sp. PCC 6803 suitable to be used as a photoautotrophic biotechnological platform (Santos-Merino et al., 2019; Vermaas, 1996).

**Scheme 1:**
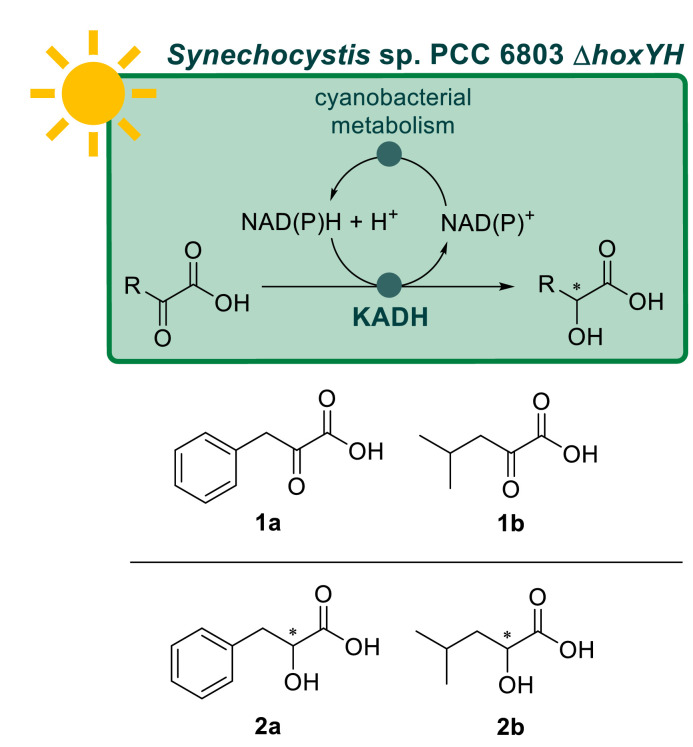
Reduction of the of the α-keto acids, phenylpyruvic acid **1a** and 4-methyl-2-oxovaleric acid **1b**, to the chiral α-hydroxy acids, phenyllactic acid **2a** and 2-hydroxy-4-methylvaleric acid **2b**, using *Synechocystis* sp. PCC 6803 Δ*hoxYH* heterologously expressing keto acid dehydrogenases. The required reduced NAD(P)H is regenerated by the metabolism.

To investigate strengths and limitations of cyanobacterial α-keto-acid reduction, we introduced α-keto acid dehydrogenases (KADH) in *Synechocystis* sp. PCC 6803 wild type, as well as a markerless deletion mutant (Δ*hoxYH*) lacking the functional Hox electron sink. In light-fluctuating conditions of natural habitats, the photosynthetic apparatus and cell metabolism of cyanobacteria are protected from an overflow of reactive electrons by action of several natural electron sinks such as the flavodiiron proteins, the type I NADH dehydrogenase (NDH-1) (Santana-Sanchez et al., 2019) and the bidirectional hydrogenase Hox (Tamagnini et al., 2007). However, these electron sinks are not needed in controlled laboratory conditions and enhanced activity of a heterologous ene-reductase and a cytochrome P450 were demonstrated upon the deletion of flavodiiron in *Synechocystis* sp. PCC 6803 (Assil-Companioni et al., 2020) and NDH-1 in *Synechococcus* sp. PCC 7002 (Berepiki et al., 2018), respectively. The Δ*hoxYH* was originally constructed as a chassis for hydrogen production and characterized as a robust photoautotrophic host due to adjusted metabolism (Pinto et al., 2012). The reduction of stereodiverse α-keto acids, phenylpyruvic acid **1a** and 4-methyl-2-oxovaleric acid **1b**, to the corresponding α-hydroxy acids **2a-b**, was chosen as the benchmark reaction (Scheme 1). α-Hydroxy acids can be found in various natural products, as well as in pharmaceutical and plant-protection agents (Coppola and Schuster, 1997; Hammerer et al., 2018). Furthermore, they are essential constituents of depsipeptides where they act as mimetics for the corresponding natural amino acids like **2a**-**b** for phenylalanine and leucine, respectively (Bunyajetpong et al. 2006; Lemmens-Gruber et al., 2009; Tripathi et al., 2010; Scherkenbeck et al., 2012).

## 2 Materials and methods

Further methods, including ‘General, kits and instruments’, ‘DNA sources and assembly’, ‘*Synechocystis* sp. PCC 6803 transformation and transformants confirmation’, ‘Expression in *E. coli*’, ‘Growth curves and chlorophyll *a* content’ can be found in the supplementary information (SI).

### 2.1 Cultivation of Synechocystis sp. PCC 6803

*Synechocystis* sp. PCC 6803 cells were cultivated in BG11 medium (Stanier et al., 1971) supplemented with HEPES (5 mM, pH 8) at 30°C under a 16 h light (Lumitronix, Powerbar V3 cool white 5700K)/8 h dark regimen. Mutants harbouring pSEVA251 plasmids were grown in the presence of kanamycin (seed 100 µg mL^−1^, working culture 50 µg mL^−1^). Seed cultures were grown in BG11 (30 mL) in baffled 100 mL Erlenmeyer flasks under shaking at 180 rpm and illumination (30 µE m^−2^ s^−1^) for 5-9 days. Working cultures were inoculated from seed cultures to an OD_750_ of 0.1 in BG11 (150 mL), inside 250 mL gas-washing flasks and grown under 80 µE m^−2^ s^−1^ light-intensity while continuous bubbling with water-saturated air, sterilized by pumping through a venting filter (PTFE 0.2 µm pore size, Midisart 2000) until reaching an OD_750_ of 1-2 (approx. 5 days).

### 2.2 Preparation of Synechocystis sp. PCC 6803 cell lysates

Working cultures were harvested by centrifugation (4°C, 20 min, 3184 *g*), the pellet was suspended in washing buffer (10 mM potassium phosphate, pH 7.5) and centrifuged again. The pellet was suspended in potassium phosphate buffer (100 mM, pH 7.5) supplemented with protease inhibitors aprotinin (3 µM) and aminocaproic acid (1 mM) to reach a final OD_750_ of 20, and sonicated on ice (5 min, amplitude 30%, 1 sec ON, 4 sec OFF). The sonicated cells were centrifuged for 25 minutes at 17 000 *g* and 4°C and the resulting blue coloured cell lysate was decanted, stored at -20°C and used in spectrophotometric activity assay and *in vitro* biotransformations.

### 2.3 Spectrophotometric activity assay

*Synechocystis* sp. PCC 6803 cell lysate was prepared as described above. *E. coli* dry lyophilized lysate (prepared according to the Supporting Method S4, see SI), coenzymes and substrates were prepared in potassium phosphate buffer (100 mM, pH 7.5). The total protein concentrations of the cell lysate were determined with Pierce^TM^ BCA Protein Assay Kit (*Synechocystis* sp. PCC 6803 D-HicDH 172.5 µg mL^−1^, *Synechocystis* sp. PCC 6803 L-HicDH 147.5 µg mL^−1^, *E. coli* D-HicDH 4.1 µg mL^−1^, *E. coli* L-HicDH 3.5 µg mL^−1^). The lysate (10 μL, concentration as indicated) was mixed with NADH or NADPH (2 mM, 50 μL; final concentration 0.9 mM) in a 96-well plate. The reaction was started by the addition of the substrate (**1a** or **1b**, 4 mM 50 μL; final 1.8 mM), final reaction volume of 110 μL. After 3 sec of initial shaking, the decrease in absorption of NAD(P)H at 340 nm was followed for 10 min at 30°C. Activities of control reactions without the substrate were subtracted from the reaction samples.

### 2.4 General procedure for in vitro biotransformation with Synechocystis sp. PCC 6803 cell lysate

*Synechocystis* sp. PCC 6803 cell lysate (500 μL) was added to a potassium phosphate buffer solution (500 μL, 100 mM, pH 7.5) containing the substrate (20 mM) and the cofactor NADH or NADPH (1 equiv.). The substrate (**1a**) was added as a DMSO solution (0.25 M stock conc., 4% final DMSO content) to overcome its poor solubility in water. The mixture was shaken at 30°C and 120 rpm. After 16 h, reaction mixtures were acidified with aqueous HCl (110 μL, 2 M), saturated with sodium chloride and extracted twice with ethyl acetate (400 μL, 250 μL) containing *n*-decanol (10 mM) as an internal GC standard. The combined organic phases were dried over anhydrous sodium sulphate and analysed as described below.

### 2.5 General procedure for in vivo biotransformations with Synechocystis sp. PCC 6803

Working cultures were harvested by centrifugation (30°C, 45 min, 2786 *g*) and the pellet was resuspended in BG11 to reach a final OD_750_ of 20 or 40. Cell suspension (500 μL) was added to a BG11 solution (500 μL) supplemented with HEPES (200 mM, pH 7.5) containing the substrate (20 mM, final 10 mM). **1a** was added as DMSO solution (0.25 M stock conc., 4% final DMSO concentration) to overcome its poor solubility in water. The reactions were performed in 1.5 mL screw-top glass GC-vials. The vials were placed in a custom photoreactor (Winkler et al., 2021) equipped with cool white LEDs (LED stripes, 5200 K) at 600 rpm and RT under average 215 µE m^−2^ s^−1^ light intensity (photoreactor settings: frequency 100 Hz, duty range 100, duty cycle 5) or covered with aluminium foil for dark reactions. After 16 h, the reaction mixtures were worked up as described above.

### 2.6 Quantification of the cell dry weight

Working cultures grown and harvested as above were shock-frozen in liquid nitrogen, lyophilized overnight and weighed in three independent experiments for each strain. A pellet of *Synechocystis* sp. PCC 6803 wild type corresponding to an OD_750_ of 10 was equivalent to 2.24 ± 0.11 g L^−1^ CDW, of Δ*hoxYH* to 2.26 ± 0.2 g L^−1^ CDW, of Δ*hoxYH* D-HicDH to 2.24 ± 0.13 g L^−1^ CDW, Δ*hoxYH* L-HicDH to 2.20 ± 0.02 g L^−1^ CDW.

### 2.7 Analytical procedures

#### 2.7.1 Determination of conversion

The compounds were analysed as their corresponding trimethylsilyl esters. The organic phase (100 μL) was mixed with pyridine (60 μL) and BSTFA (60 μL) and heated for 1 h at 60°C and 600 rpm (**1a**/**2a**) or incubated at room temperature for 20 min (**1b**/**2b**). **1a** and **2a** were quantified using an HP-5 (30 m × 0.25 mm × 0.25 µm) column with the following temperature program: 100°C for 0.5 min, 10°C min^−1^ to 300°C, split ratio 50:1, injection volume 1 µL. Retention times: *n*-decanol 6.88 min, **1a** and **2a** 10.81 min and 9.47 min, respectively. **1b** and **2b** were quantified using a DB-1701 (30 m × 0.25 mm × 0.25 µm) column with the following temperature program: 40°C for 2 min, then 10°C min^−1^ to 180°C, split ratio 90:1, injection volume 1 µL. Retention times: *n*-decanol 14.45 min, **1b** and **2b** 12.08 min and 13.05 min, respectively. Gas chromatography was performed using an Agilent Technologies 7890A GC(FID) system.

#### 2.7.2 Determination of the enantiomeric excess

The compounds were analysed as the corresponding methyl esters. Organic phase (100 μL) was mixed with methanol (10 μL) and TMS (5 μL). Enantiomeric excess of **2a** was measured using an Rt®-BDEXse (30 m x 0.32 mm x 0.25 µm) column with the following temperature program: 60°C for 1 min, 5°C min^−1^ to 180°C, split ratio 50:1 and injection volume 1 µL. Retention times: (*R*)-**2a** and (*S*)-**2a** 20.55 min and 20.8 min, respectively. Enantiomeric excess of **2b** was measured using a Chirasil Dex-CB (25 m x 0.32 mm x 0.25 µm) column with the following temperature program: 60°C for 2 min, 3°C min^−1^ to 110°C, 10°C min^−1^ to 200°C, split ratio 50:1 and injection volume 1 µL. Retention times: (*R*)-**2b** and (*S*)-**2b** 15.33 min and 15.88 min, respectively. Gas chromatography was performed using an Agilent Technologies 7890A GC(FID) system. The enantiomeric excess (*ee*) was calculated based on the GC spectra with Eq (1)., where E1 and E2 were respective peak areas of enantiomers in the mixture.

(1) $$ee\;\left[\%\right]=\frac{\left|E1-E2\right|}{E1+E2}*100$$

For representative chromatograms of commercial references and biotransformations, as well as calibration curves, see SI (Fig. S6 – S10).

## 3 Results and discussion

In an initial screening, we tested the activity of five keto acid dehydrogenases towards the target substrates **1a** and **1b** in the presence of NADH and NADPH to determine the suitability of the enzymes to catalyze the target reaction in cyanobacteria, which provide NADPH as reducing agent (Tamoi et al., 2005). The enzymes were expressed in *E. coli* BL21(DE3) and the whole cell lyophilizates were then investigated in a spectrophotometric assay (Table S5). The best performing enzymes were a pair of enantio-complementary keto-acid dehydrogenases, namely L- and D-2-hydroxyisocaproate dehydrogenase derived from *Lactobacillus confusus* DSM 20196 (UniProtKB ID: P14295) (Schutte et al., 1984) and *Lactobacillus paracasei* DSM 20008 (UniProtKB ID: P17584) (Hummel et al., 1985), respectively (hereafter L-HicDH and D-HicDH). It is noteworthy that, in contrast to literature reports (Hummel et al., 1985; Schutte et al., 1984; Busto et al., 2016, 2014; Gourinchas et al., 2015), where both enzymes were described as NADH-dependent, they displayed reasonable activity, although reduced, also in the presence of NADPH (Table 1). Furthermore, in end-point biotransformations supplied with NADPH, good conversions in the range of 84% to 90% and perfect stereoselectivity towards both, the (*R*)- or the (*S*)-products, depending on the applied enzyme, were reached (Table S6).

**Table 1:** Specific activity of L- and D-HicDH cell lysates from *E. coli* and *Synechocystis* sp. PCC 6803 in the presence of NAD(P)H, measured photometrically as decrease in NAD(P)H absorbance at 340 nm.

enzyme	substrate	cofactor	specific activity [U mg^−1^]^a^	
			*E. coli*^b^	*Synechocystis* sp. PCC 6803 Δ*hoxYH*^c^
L-HicDH	**1a**	NADH	130.8 ± 7.5	0.3 ± 0.1
		NADPH	12.8 ± 1.0	0.0 ± 0.1
	**1b**	NADH	31.5 ± 2.0	0.1 ± 0.1
		NADPH	12.8 ± 2.9	0.1 ± 0.1
D-HicDH	**1a**	NADH	520.1 ± 13.6	14.1 ± 0.4
		NADPH	30.4 ± 2.6	1.1 ± 0.1
	**1b**	NADH	31.8 ± 0.1	0.8 ± 0.1
		NADPH	6.2 ± 4.5	0.3 ± 0.0

Reaction conditions: substrate (1.8 mM), NADH or NADPH (0.9 mM), potassium phosphate buffer (100 mM, pH 7.5, 30°C). Average and standard deviation of technical triplicates, background activity of control reactions without substrate subtracted.

a Units per mg total protein

b L-HicDH 0.3 µg mL^−1^, D-HicDH 0.4 µg mL^−1^ total protein

c L-HicDH 13.8 µg mL^−1^, D-HicDH 15.7 µg mL^−1^ total protein

These results motivated us to express both HicDH enzymes in *Synechocystis* sp. PCC 6803. Synthetic genes encoding L- and D-HicDH, codon optimized for expression in the cyanobacterium (for details see Supporting Information), were cloned into the pET21a(+) vector and overexpressed in *E. coli* BL21(DE3). Expression of active enzymes using the synthetized genes was confirmed by remeasuring the activity towards **1b** (Fig. S1). The codon optimized genes were then subcloned in the pSEVA251 replicative vector (Silva-Rocha et al., 2013), under the regulation of different synthetic medium-strength constitutive promoters (P*_trc.x.tetO2_* or P*_trc.x.lacO_*) (Ferreira et al., 2018) and the RBS BBa_B0030 from the Registry of Standard Biological Parts (Englund et al., 2016; Registry of Standard Biological Parts parts.igem.org). The construct with D-HicDH under the control of the P*_trc.x.lacO_* promoter showed notable genetic instability due to which the plasmid with the correct sequence could not be produced in a sufficient yield for cyanobacterial transformation. pSEVA251_P*_trc.x.lacO_::L-HicDH*, pSEVA251_P*_trc.x.tetO2_::L-HicDH* and pSEVA251_P*_trc.x.tetO2_::D-HicDH* were introduced in the *Synechocystis* sp. PCC 6803 wild type and the Δ*hoxYH* strain, however only two transformants, both in the Δ*hoxYH* genetic background, were successfully identified: the L-HicDH under the control of P*_trc.x.lacO_* promoter (plasmid pSEVA251_P*_trc.x.lacO_::L-HicDH*; hereafter *S.* L-HicDH) and D-HicDH under the control of the P*_trc.x.tetO2_* promoter (plasmid pSEVA251_P*_trc.x.tetO2_::D-HicDH*; hereafter *S.* D-HicDH). The troublesome cloning suggests a harmful effect of constitutive expression of the KADHs on the host cell metabolism. However, growth rates and chlorophyll *a* content of the strains harbouring the HicDHs were comparable to the *Synechocystis* sp. PCC 6803 wild-type and Δ*hoxYH* (Fig. S3). Additionally, even though the P*_trc.x.lacO_* is reported as a stronger promoter than P*_trc.x.tetO2_* (Ferreira et al., 2018), the fact that L-HicDH was only expressed under the control of P*_trc.x.lacO_* supports reports on greater complexity of the regulation in *Synechocystis* sp. PCC 6803, depending also on genetic context (Thiel et al., 2018).

Measuring the activity of cyanobacterial cell lysates in a spectrophotometric assay using substrates **1a** and **1b** confirmed active expression of D-HicDH. However, the activity of L-HicDH was detectable only with **1a** in the presence of NADH, which was also the preferred combination when expressed in *E. coli* (Table 1). In addition, the activity of the cyanobacterial cell lysate was generally more than one order of magnitude lower compared to *E. coli* lysate. The difference in activity of cyanobacterial and *E. coli* enzyme preparations suggest that the amount of the active D-HicDH expressed per weight of total soluble protein was roughly 30-fold lower in the cyanobacteria while this difference in case of L-HicDH was more than 400-fold. The lesser amount of the target enzyme reflects our choice of a regulatory system in *Synechocystis*, that is based on a relatively low-level constitutive expression in order to minimize the metabolic burden on the cyanobacterial host, as compared to the strong inducible system that was used in *E. coli*.

**Fig. 1:**
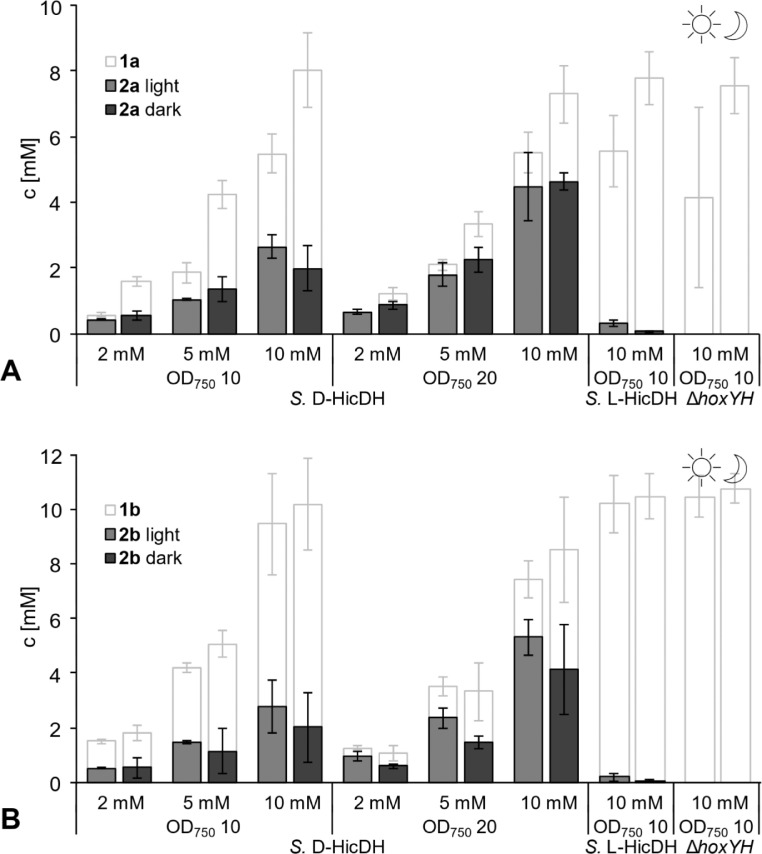
*In vivo* biotransformations of (**A**) phenylpyruvic acid (**1a**) and (**B**) 4-methyl-2-oxovaleric acid (**1b**) (2-10 mM) using cyanobacterial cells. Substrate loading and cell loading of *S.* D-HicDH were investigated. End-point measurements, reaction time 16 h. Enantiomeric excess of reactions with *S.* D-HicDH was >99% for (*R*)-phenyllactic acid (*R*)-**2a** and (*R*)-2-hydroxy-4-methylvaleric acid (*R*)-**2b** and was not determined for compounds under 0.5 mM. Left = light, right = dark. Average and standard deviation of three independent experiments.

We investigated whether the living cyanobacterial cells, upon illumination with white light, could provide the reducing equivalents needed for the ketoreduction of **1a**-**b** (Fig 1AB). Remarkably, the *S.* D-HicDH at OD_750_ of 10 was able to convert 26% of **1a** and 28% of **1b** with perfect stereoselectivity to the corresponding optically pure products (*R*)-**2a** or (*R*)-**2b** (ee >99%). Interestingly, when the substrate loading was increased from the initial 2 mM to 5 and 10 mM, the percentage of formed product stayed in the same range, effectively demonstrating a higher productivity while retaining the perfect ee of >99% (Fig. 1AB and Fig. S4). This trend might be explained by a limited uptake of the polar substrates, which is increased at a higher substrate concentration. The overall conversion could be even boosted by increasing the cell density to an OD_750_ of 20, reaching 45% at a concentration of 10 mM of **1a** and 53% at 10 mM of **1b**. In contrast, the *S.* L-HicDH at OD_750_ of 10 was less active, as it only converted less than 0.3 mM of 10 mM substrate to (*S*)-**2a** or (*S*)-**2b**.

**Fig 2:**
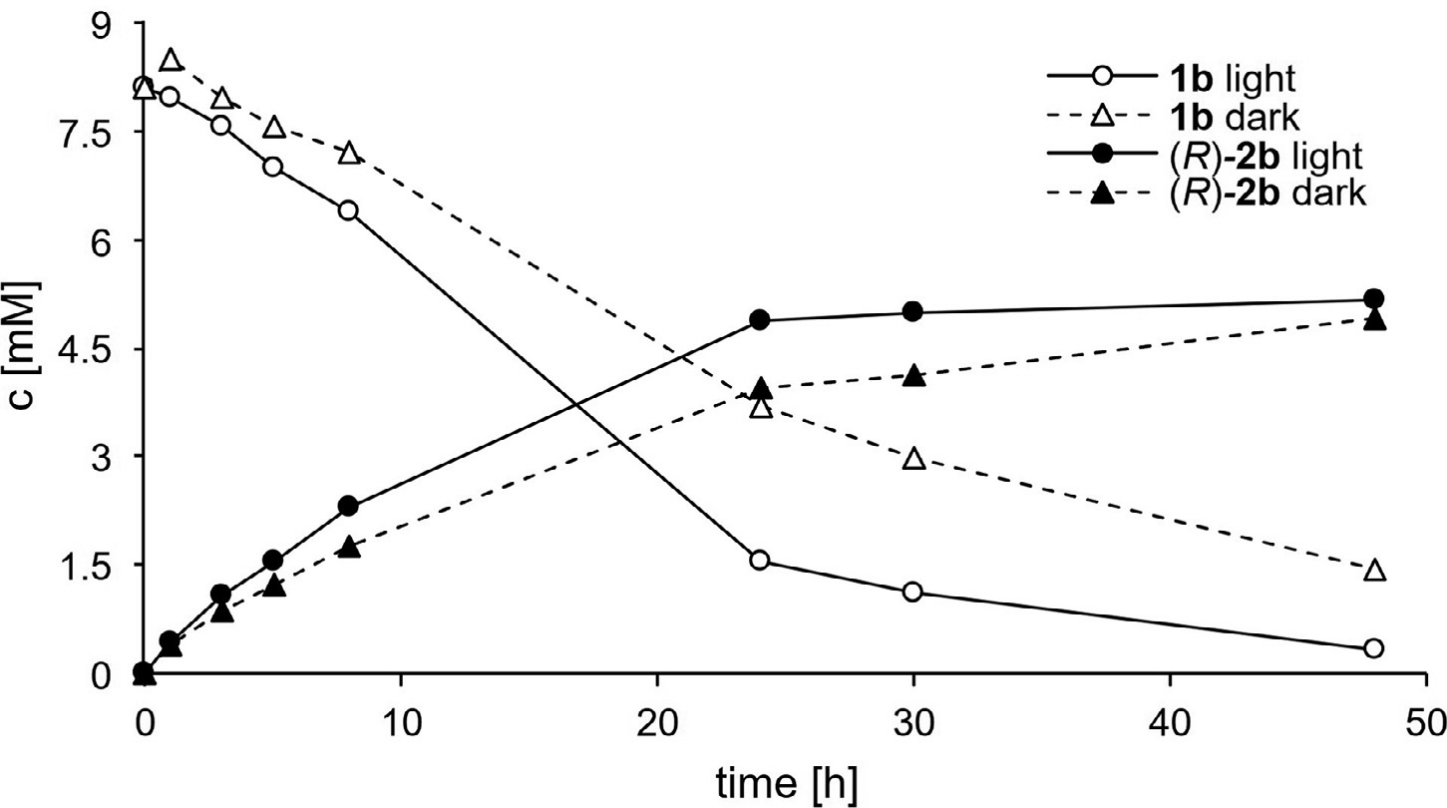
Time course of the *in vivo* biotransformation of **1b** (10 mM) by cyanobacterium *S.* D-HicDH (OD_750_ 10).

Another option to increase the conversion by *S.* D-HicDH at OD_750_ of 10 was by increasing the reaction time (Fig. 2). Under light, 49% of **1b** was converted to (*R*)-**2b** after 24 hours and after 48 hours the conversion remained at 52%. Still, **1b** continued to be depleted at a constant rate even between 24 and 48 hours. Initially, the dark reaction proceeded at a slower pace, reaching 39% product after 24 hours, but catching up with the light reaction after 48 hours at 49%.

The amount of α-hydroxy acids **2a**-**b** that was produced with *S.* D-HicDH under irradiation with white light, was comparable to the results obtained under dark conditions (Fig. 1AB, Fig. 2). As the photosynthetic machinery directly produces NADPH and is therefore considered the major reducing equivalent (Park and Choi, 2017), the comparable activity in the dark suggests that reducing equivalents may also originate from other parts of the metabolism, such as glycolysis (upon glycogen degradation) and in the form of NADH. Therefore, the presented process is light-dependent in the sense that the growth of the biocatalyst and provision of reducing equivalents are autotrophic, but light is not a direct driving force of the targeted activity, as it can also take place in the dark (Löwe et al., 2018). A similar dark reaction has been found previously when imine reductases were heterologously expressed in *Synechocystis* sp. PCC 6803 (Büchsenschütz, 2020).

No background keto-acid dehydrogenase activity of the Δ*hoxYH* was detected, however, we observed lower recovery, particularly with **1a** as substrate. The cells did not demonstrate activity towards *rac-***2a or** *rac-***2b** (Fig. S5A); therefore, the mass balance loss can be attributed to **1a** and **1b**. Partly, the loss of **1a** is due to spontaneous decomposition, as less than 80% of **1a** is recovered in both light and dark conditions upon incubation of **1a** only in the reaction buffer for 16 h (Fig. S5B). However, in the presence of cyanobacterial cells (Fig. 1A), the recovery was always notably lower under irradiation, regardless of the presence of recombinant enzyme. This loss of substrate may not be surprising knowing that phenylpyruvate **1a** is one of the central metabolites in phenylalanine metabolism of *Synechocystis* sp. PCC 6803*,* that is connected to other pathways such as biosynthesis of tropane, piperidine and pyridine alkaloids (pathway syn00360 in the KEGG, Kyoto Encyclopedia of Genes and Genomes (Kanehisa, 2000)). Unlike the targeted keto-reduction, the substrate degradation showed clear light-dependence. Similarly, α-ketoisocaproic acid **1b** is also a natural metabolite in branched amino acid biosynthesis (KEGG pathway syn00290) but it generally has less of a central role, therefore the side-reactivity is less pronounced.

**Fig. 3:**
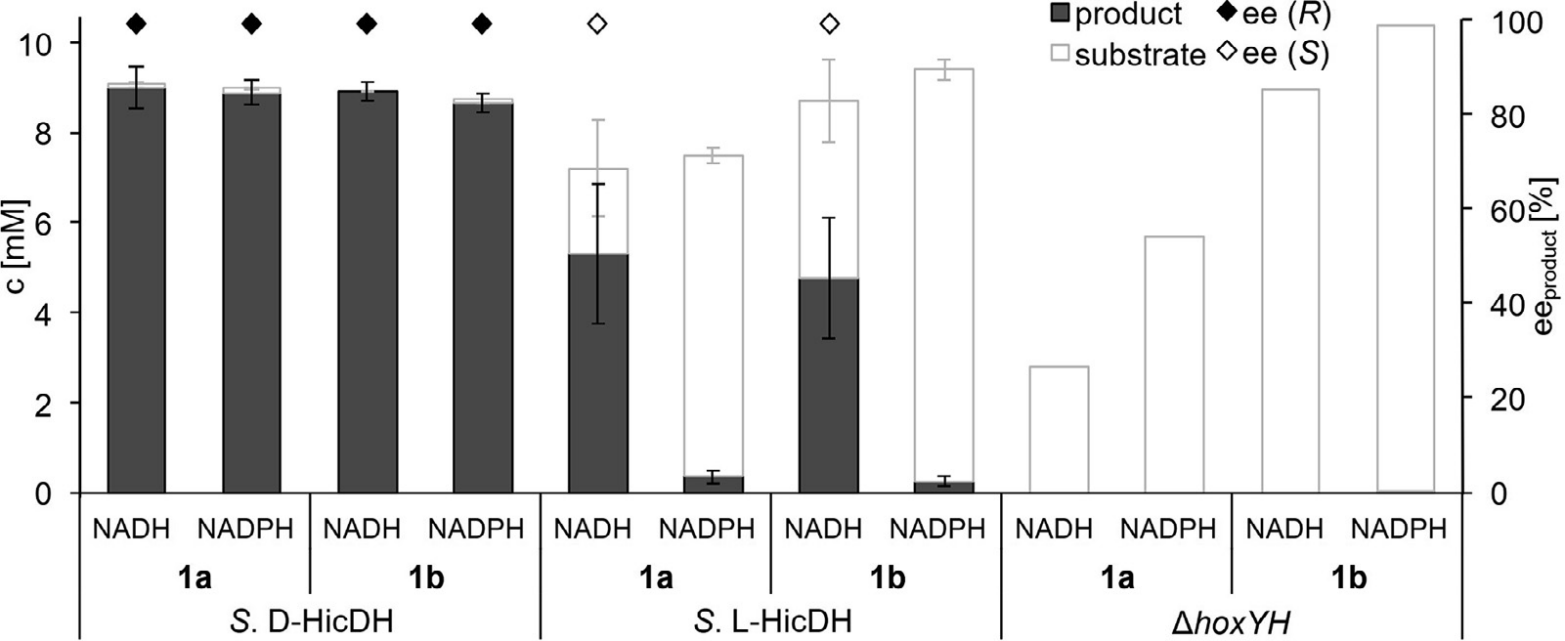
*In vitro* biotransformations of phenylpyruvic acid (**1a**) and 4-methyl-2-oxovaleric acid (**1b**) (10 mM) using cyanobacterial cell lysates in presence of 1 equivalent of NAD(P)H. Enantiomeric excess for the corresponding products was not determined for concentrations below 0.5 mM. Error bars correspond to standard deviation of biological triplicates.

When cyanobacterial cell lysates corresponding to an OD_750_ of 10 were exogenously supplied with an equivalent of NAD(P)H, nearly 90% conversion of both substrates by *S.* D-HicDH lysate was observed regardless of the cofactor, demonstrating the application of the strain for the photosynthetic production of the target enzymes (Fig. 3). This also suggests that the limiting factor of *in vivo* biotransformations is not the amount of D-HicDH expressed but rather the availability of reduced cofactor NADH or the transport of the substrate into the cell. On the other hand, *S.* L-HicDH lysate converted roughly 50% of **1a-b** in the presence of NADH and <5% in the presence of NADPH, confirming our hypothesis that for this strain, the amount of active enzyme is another limiting factor. Control reactions with the cyanobacterial lysate free of the recombinant enzymes (Δ*hoxYH*) resulted in decrease of substrate concentrations. The loss of **1a** was especially pronounced in reactions supplied with NADH and, to a lesser extent, NADPH. This result suggests that some of the competing reactions are dependent of NAD(P)H and explains their light-dependency *in vivo*.

In conclusion, the keto acid dehydrogenases D-HicDH and L-HicDH, initially expressed in *E. coli*, showed reasonable activity also with NADPH although NADH is the preferred cofactor. Both enzymes were successfully expressed under constitutive regulation only in the *Synechocystis* sp. PCC 6803 Δ*hoxYH* mutant and their activity was demonstrated, albeit the *S.* L-HicDH showed very poor expression and consequently activity. We hypothesize that the interference of heterologous HicDHs in the metabolic pathways of *Synechocystis* sp. PCC 6803 imposed a heavy metabolic burden to the host organism which only the Δ*hoxYH* mutant was able to alleviate, indicating a greater degree of metabolic plasticity of this mutant.

The cyanobacterium expressing the D-HicDH was able to convert up to 46% of **1a** and 53% of **1b** at a substrate concentration of 10 mM giving the corresponding α-hydroxy acid in optically pure form (*ee* >99%). One limitation in the cell's productivity might be the uptake of the polar α-keto acid substrates at the conditions used.

Experiments in the dark and with the cell lysates indicated that the HicDHs most likely consumed NADH from the cell's metabolism instead of the photosynthetically produced NADPH, which can be attributed to the enzymes’ preference for NADH. Nevertheless, as the cells grow only using CO_2_ as carbon source and light as energy source, the overall process is still light dependent. The use of the photosynthetically produced enzymes in form of a lysate with externally added NADH allowed to reach conversions of up to 90% for **1a** and 89% for **1b**.

This study indicates that the observed cofactor promiscuity of the studied enzymes towards NADPH is not sufficient for exploiting the light-driven cyanobacterial metabolism, which might be mitigated by using strictly NADPH-dependent enzymes or by increasing internal NADH supply by co-expressing a transhydrogenase (Angermayr et al., 2012; Niederholtmeyer et al., 2010) and engineering the electron transport (Meng et al., 2021). Moreover, the activity may be improved via inducible regulation of enzyme expression. Furthermore, the reaction might be additionally tuned by supplying the substrates in a form that can more easily cross the cell wall (e.g. as esters) or by providing a CO_2_ rich environment for the whole cell catalysts.

Overall, we demonstrated the successful inducer-free expression of D-HicDH and L-HicDH from replicative plasmids in *Synechocystis* sp. PCC 6803 Δ*hoxYH* mutant, confirming its robustness as a host in comparison to the wild-type.

## Declaration of competing interest

The authors declare that they have no known competing financial interests or personal relationships that could have appeared to influence the work reported in this paper.

## Appendix Supplementary materials

Image, application 1
