# Supplementary material for: Expression and activity of heterologous hydroxyisocaproate dehydrogenases in Synechocystis sp. PCC 6803 ΔhoxYH
Source: Eng Microbiol. 2021 Nov 26;2(1):100008. doi: 10.1016/j.engmic.2021.100008 (PMC11610949; doi:10.1016/j.engmic.2021.100008)
Supplement: Supplementary file 1 [file mmc1.pdf]

Supporting Information

Expression and activity of heterologous hydroxyisocaproate dehydrogenases in *Synechocystis* sp. PCC 6803 Δ*hoxYH*

Valentina Jurkaš[a], Christoph K. Winkler[a], Silvan Poschenrieder[a], Paulo Oliveira[b,c,d], Catarina C. Pacheco[b,c], Eunice A. Ferreira[b,c,e], Florian Weissensteiner[a], Piera De Santis[f], Selin Kara[f], Robert Kourist[g], Paula Tamagnini[b,c,d*], Wolfgang Kroutil[a,h,i*]

[a]*Institute of Chemistry, University of Graz, NAWI Graz, Heinrichstrasse 28, 8010 Graz, Austria.*

[b]*i3S – Instituto de Investigação e Inovação em Saúde, Universidade do Porto, 4200-135 Porto, Portugal.*

[c]*IBMC – Instituto de Biologia Molecular e Celular, Universidade do Porto, 4200-135 Porto, Portugal.*

[d]*Departamento de Biologia, Faculdade de Ciéncias, Universidade do Porto, 4169-007 Porto, Portugal.*

[e]*ICBAS – Instituto de Ciências Biomêdicas Abel Salazar, Universidade do Porto, 4050-313 Porto, Portugal.*

[f]*Aarhus University, Department of Engineering, Biological and Chemical Engineering Section, Biocatalysis and Bioprocessing Group, Gustav Wieds Vej 10, DK 8000 Aarhus, Denmark.*

[g]*Institute of Molecular Biotechnology, Graz University of Technology, 8010 Graz, Austria.*

[h]*Field of Excellence BioHealth – University of Graz, 8010 Graz, Austria.*

[i]*BioTechMed Graz, 8010 Graz, Austria.*

*corresponding authors: wolfgang.kroutil@uni-graz.at and pmtamagn@ibmc.up.pt

Table of Contents

Supporting Methods **3**

S1 General, kits and instruments 3

S2 DNA sources and assembly 4

S3 *Synechocystis* sp. PCC 6803 transformation and transformants confirmation 5

S4 Expression in *E. coli* BL21(DE3) 6

S5 Growth curves and chlorophyll *a* content of *Synechocystis* sp. PCC 6803 working cultures 7

Supporting Tables **8**

Table S1. List of plasmids used in this study. 8

Table S2. List of primers used in this study. 9

Table S3. List of *E. coli* strains used in this study 10

Table S4. List of *Synechocystis* sp. PCC 6803 strains used in this study. 10

Table S5. Specific activity of keto acid dehydrogenases in form of lyophilized whole cells in the presence of NAD(P)H, measured photometrically as decrease in NAD(P)H absorbance at 340 nm. 11

Table S6. Biotransformation of α-keto acids 1a-b to α-hydroxy acids 2a-b, catalysed by lyophilized whole *E. coli* cells expressing L- or D-HicDH, in the presence of NADPH. 11

Supporting Figures **12**

Expression and activity of HicDHs in *E. coli* harbouring the genes codon optimized for *Synechocystis* sp. PCC 6803 12

Colony PCR of *S.* D-HicDH and *S.* L-HicDH 12

Growth curves of *Synechocystis* sp. PCC 6803 strains 13

In vivo product formation 14

In vivo control reactions 15

Representative chromatograms 16

Calibration curves 20

DNA and amino acid sequences **21**

D-HicDH 21

L-HicDH 22

References **23**

Supporting Methods

S1 General, kits and instruments

General reagents, substrates and solvents were purchased and used as supplied from Sigma-Aldrich (Merck KGaA), Thermo Fisher Scientific, Fluka, Lancaster and Roth. Restriction enzymes and buffers were from Thermo Scientific. Ligation was performed with the T4 ligase from Thermo Scientific. PCR was performed with the Thermo Scientific Phusion High-Fidelity DNA Polymerase and buffer on an Eppendorf Nexus Gradient Mastercycler. Colony PCR was performed with GoTaq Flexi in GoTaqFlexi Green buffer (Promega) or DreamTaq Green PCR Master Mix (Thermo Scientific). Gel extraction, DNA purification and plasmid isolation were performed with the QIAquick gel extraction kit and the QIAGEN Miniprep kit or NZYGelPure gel extraction kit and the NZYMiniprep kit from NZYtech. Optical densities of *E. coli* cell cultures and enzyme concentrations of enzyme preparations were determined on an Eppendorf Biophotometer Plus. Optical densities and chlorophyll *a* content of *Synechocystis* sp. PCC 6803 cell cultures were measured on a Cary 60 UV-Vis photometer from Agilent Technologies. DNA was quantified on a NanoDrop ND-1000 spectrophotometer (NanoDrop Technologies, Inc.) or an Eppendorf Biophotometer Plus. SDS-polyacrylamide gel electrophoresis was performed with Gene Script ExpressPlus™ page gels. Cell disruption was carried out with a BRANSON Digital Sonifier. Spectrophotometric measurements were done on a Spectra Max and M2 plate reader from Molecular Devices and analysed in Soft Max. 4220.0 mM^{-1} cm^{-1} was used as the extinction coefficient of NAD(P)H.

S2 DNA sources and assembly

Vectors for expression of D-HicDH from *Lactobacillus confusus* DSM 20196 (UniProtKB ID: P14295) and L-HicDH from *Lactobacillus paracasei* DSM 20008 (UniProtKB ID: P17584) in *E.* coli previously reported (Gourinchas et al., 2015) were available in the in-house plasmid collection and were transformed into chemically competent *E. coli* BL21(DE3).

For expression in cyanobacteria, the coding sequences (CDS) of D-HicDH and L-HicDH were codon-optimized for expression in *Synechocystis* sp. PCC 6803 using Gene Designer 2.0 (Villalobos et al., 2006), synthesized by GeneArt (Invitrogen) and cloned into pET21a(+) using NdeI and XhoI restriction sites. *E. coli* NEB 5-alpha were used for cloning, according to the manual with ampicillin (100 µg mL^{-1}) as selective marker. The sequences of transformants were confirmed by sequencing with T7 primers. These genes were then subcloned in the pSEVA251 replicative vector (Silva-Rocha et al., 2013), under the regulation of different medium-strength constitutive promoters ($P_{trc.x.tetO2}$ or $P_{trc.x.lacO}$) (Ferreira et al., 2018) and the RBS BBa_B0030 from the Registry of Standard Biological Parts (Englund et al., 2016), using the BioBrick Standard Assembly method (parts.igem.org). The genes, as well as promoter-RBS sequences (Ferreira et al., 2018) were amplified by PCR with primers flanked by the BioBrick^{TM} prefix or suffix (parts.igem.org), using Phusion High-Fidelity DNA polymerase, according to the manufacturer's instructions. The PCR products were purified from agarose gel, promoter-RBS amplicon was digested with EcoRI and SpeI and the CDS amplicon with XbaI and PstI. Constructs were assembled directly in a pSEVA251 shuttle vector (obtained from the 'Standard European Vector Architecture' repository) (Silva-Rocha et al., 2013), digested with EcoRI and PstI. Both inserts were simultaneously ligated with the vector (Scheme S1) using a T4 DNA ligase. Ligation mixtures were transformed into chemically competent *E. coli* XL1-Blue and grown on LB agar plates supplemented with kanamycin (50 µg mL^{-1}) as a selective marker. The correct assembly of the generated constructs was confirmed by PCR, restriction analysis and plasmid sequencing with PS1 and PS2 primers. All the plasmids used in this study are listed in Table S1, and the sequences of primers in Table S2.

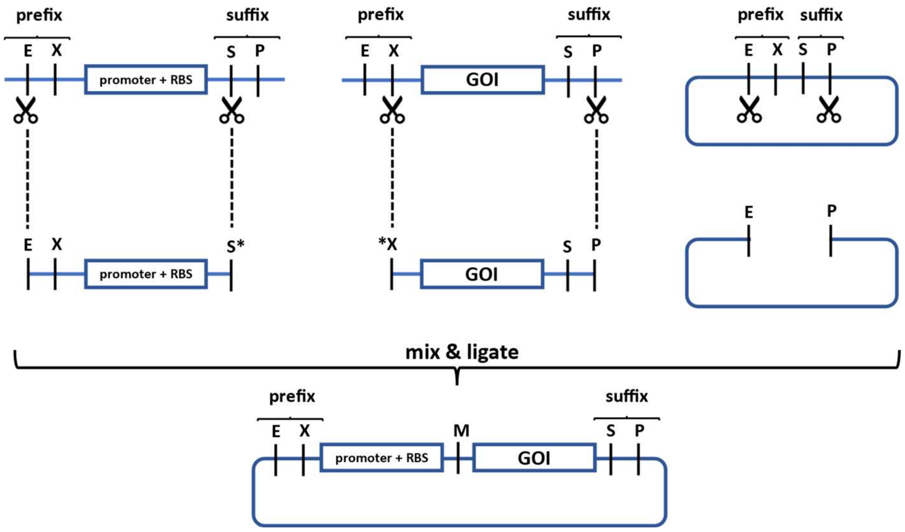

Scheme S1. The BioBrick Assembly method (parts.igem.org). Upstream regulatory elements are digested with EcoRI and SpeI and the gene of interest with XbaI and PstI. SpeI and XbaI generate identical overhangs that ligate creating a mixed site (scar) that cannot be cut with any of the enzymes. E, EcoRI; X, XbaI; S, SpeI; P, PstI, M, mixed site; GOI, gene of interest; RBS, ribosomal binding site.

S3 *Synechocystis* sp. PCC 6803 transformation and transformants confirmation

Wild-type and mutants of the cyanobacterium *Synechocystis* sp. PCC 6803 (Table S4) were maintained in BG11 medium (Allen, 1968; Stanier et al., 1971). For solid BG11, the medium was supplemented with 1.5% (wt/vol) noble agar (Difco), 0.3% (wt/vol) sodium thiosulfate and TES-KOH buffer (10 mM, pH 8.2). For the selection and maintenance of mutants, BG11 medium was supplemented with kanamycin (Kan, 10–500 µg mL^{-1}).

The pSEVA251 plasmids carrying assembled constructs (Table S1) were introduced into *Synechocystis* sp. PCC 6803 by electroporation, as described previously (Ferreira et al., 2018; Ludwig et al., 2008). *Synechocystis* sp. PCC 6803 cultures were grown in 50 mL BG11 at 30 °C under a 12 h light/12 h dark regimen from OD_{730} of 0.1 until a final OD_{730} of 0.5 – 0.6. Cells were collected by centrifugation at 4470 *g* for 10 min and washed three times with HEPES buffer (10 mL, 1 mM, pH 7.5). The cells were then suspended in HEPES buffer (1 mL) and 60 µL aliquots were mixed with plasmid DNA (1 µg), transferred to electroporation cuvettes (2 mm gap electroporation cuvettes with caps, pre-sterilized, individually sealed, Molecular BioProducts) and electroporated using a Gene Pulser™ (Bio-Rad). The capacitor was set to 25 µF, the resistor to 400 Ω and potential to 2.5 V for a time constant of 9 ms with an electric field of 12 kV cm^{-1}. Immediately after the electric pulse, cells were transferred to fresh BG11 medium (400 µL) and spread onto Immobilon-NC membranes (0.45 µm pore size, 82 mm, Merck Millipore) resting on solid BG11 plates incubated at 30 °C, under a continuous low-light (shaded with a piece of paper) regimen for 24 h. Then, membranes were transferred to solid BG11 plates supplemented with kanamycin (10 µg mL^{-1}) and incubated in the conditions described above. The membranes were sequentially transferred in one-week intervals to solid BG11 plates supplemented with kanamycin (up to 500 µg mL^{-1}).

The presence of replicative plasmids in *Synechocystis* sp. PCC 6803 was confirmed by colony PCR with DreamTaq DNA Polymerase using specific primers (Table S2). To verify the sequence of the construct in *Synechocystis*, DNA was extracted using 2 mL of culture that were centrifuged at 14 000 *g* for 2 min. Then, the cell pellet was suspended in nuclease-free water (200 µL) and 2 spatula tips of 0.25-0.30 mm diameter glass beads were added. Cells were disrupted by two cycles of vortexing for 1 min with incubation on ice for 1 min in between. The suspension was centrifuged at 14 000 *g* for 2 min, and the DNA containing supernatant was transferred to a new tube and quantified. The PCR was performed using Phusion High-Fidelity Polymerase, according to manufacturer's instructions. The PCR products were purified from agarose gel and sequenced. Table S4 lists all the *Synechocystis* sp. PCC 6803 strains used in this study.

S4 Expression in *E. coli* BL21(DE3)

Plasmids isolated from *E. coli* NEB 5-alpha (Table S1) were transformed into chemically competent *E. coli* BL21(DE3). Single colonies were picked, grown overnight in LB-medium (20-30 mL; 37 °C; 120 rpm) supplemented with antibiotic (ampicillin, 100 µg mL^{-1} or kanamycin, 50 µg mL^{-1}, final concentrations) according to Table S1, and used for the inoculation of sterile LB-medium supplemented with ampicillin (100 µg mL^{-1}) (pEG220, pEG221,pET21a(+)_co_L-HicDH and pET21a(+)_co_D-HicDH) or TB-medium supplemented with kanamycin (50 µg mL^{-1}) (pEG479, pEG480, pEG493). The flasks were incubated at 37 °C and 120 rpm until an OD_{600} of 0.6 was reached, then protein expression was induced by adding IPTG (1 mM for pEG220, pEG221, pEG479, pEG480, pEG493; 0.5 mM for plasmids carrying genes codon optimized for *Synechocystis* sp. PCC 6803 pET21a(+)_co_L-HicDH and pET21a(+)_co_D-HicDH). The cultures were further incubated overnight at 25 °C (pEG220 and pEG221) or 20 °C (pET21a(+)_co_L-HicDH and pET21a(+)_co_D-HicDH, pEG479, pEG480, pEG493) and 120 rpm. To harvest the cells, cultures were centrifuged at 3184 *g*, 20 min, 4 °C, the cell pellet suspended in wash buffer (1 - 2.5 g cells per 10 mL phosphate buffer, 10 mM, pH 7), and then centrifuged again under the same conditions.

To prepare cell lysates (pEG 220 and pEG221), cell pellets were suspended in KPi (100 mM, pH 7.5) and sonicated on ice (5 min, amplitude 30%, 1 sec ON, 4 sec OFF). The sonicated cells were centrifuged for 25 minutes at 17 000 *g* and 4 °C. The rest of the supernatant (cell-free extract) was shock-frozen (liquid nitrogen) inside a round bottom flask, lyophilized, and stored at -20 °C.

Alternatively, to prepare whole cell lyophilizates, the cell pellets were suspended in a small volume of wash buffer (5-10 mL), transferred to a round bottom flask, shock frozen in liquid nitrogen and lyophilized overnight.

S5 Growth curves and chlorophyll *a* content of *Synechocystis* sp. PCC 6803 working cultures

The OD_{750} and the amount of chlorophyll *a* for each strain were determined from samples originating from at least three independent cultivations under standard growth conditions for working cultures, each measured in triplicates.

The chlorophyll *a* was determined as described (Meeks and Castenholz, 1971). A sample of the cell culture (100 µL) was mixed with cold methanol (900 µL) and incubated in darkness at 4 °C 2-3 hours to overnight. Then, the samples were centrifuged for 3 min at 14 000 *g* and the absorption of the supernatant was measured at 665 nm. The amount of chlorophyll *a* was determined using the extinction coefficient $\varepsilon = 78.74\ L\ g^{-1}\ cm^{-1}$ according to Eq. S1, where the dilution factor corresponds to 10.

$$\textbf{Eq. S1}\quad Chl\ a\ \left[\frac{\mu g}{mL}\right] = A665 * 12.7 * dilution\ factor$$

Supporting Tables

Table S1. List of plasmids used in this study.

Plasmid	Description	Selection marker	Reference/source
pET21a(+)	Vector for inducible expression from T7 promoter in *E. coli.*	Amp	-
pEG220	CDS of L-HicDH from *Lactobacillus confusus* DSM 20196 in pET21a(+).	Amp	(Gourinchas et al., 2015)
pEG221	CDS of D-HicDH from *Lactobacillus paracasei* DSM 20008 in pET21a(+).	Amp	(Gourinchas et al., 2015)
pEG479	CDS of Lactate dehydrogenase variant from *Bacillus stearothermophilus* (mutations I37K; D38S; F16Q; C81S; N85R) in pET28a(+).	Kan	(Bur et al., 1989; Flores and Ellington, 2005; Holmberg et al., 1999)
pEG480	CDS of D-2-Hydroxy acid dehydrogenase from *Haloferax mediterranei* pET28a(+).	Kan	(Domenech and Ferrer, 2006)
pEG493	CDS of YiaE from *E. coli* K12 in pET28a(+).	Kan	(Yun et al., 2005)
pET21a(+)_co_L-HicDH	CDS of L-HicDH codon optimized for expression in *Synechocystis* sp. PCC 6803 in pET21a(+).	Amp	This work
pET21a(+)_co_D-HicDH	CDS of D-HicDH codon optimized for expression in *Synechocystis* sp. PCC 6803 in pET21a(+).	Amp	This work
pSB1A3 _$P_{trc.x.tetO2}$_B0030	Synthetic promoter $P_{trc.x.tetO2}$ and the BioBrick RBS BBa_B0030 in pSB1A3 plasmid.	Amp	(Ferreira et al., 2018)
pSB1A2 _$P_{trc.x.lacO}$_B0030	Synthetic promoter $P_{trc.x.lacO}$ and the BioBrick RBS BBa_B0030 in pSB1A2 plasmid.	Amp	(Ferreira et al., 2018)
pSEVA251	Replicative shuttle plasmid, for *E.coli* and *Synechocystis*.	Kan	(Silva-Rocha et al., 2013) / Standard European Vector Architecture Repository
pSEVA251_ $P_{trc.x.lacO}$::*L-HicDH*	CDS of L-HicDH codon optimized for expression in *Synechocystis* sp. PCC 6803 under the control of the synthetic promoter $P_{trc.x.lacO}$ and the RBS BBa_B0030 in pSEVA251 plasmid.	Kan	This work
pSEVA251_ $P_{trc.x.tetO2}$::*D-HicDH*	CDS of D-HicDH codon optimized for expression in *Synechocystis* sp. PCC 6803 under the control of the synthetic promoter $P_{trc.x.tetO2}$ and the RBS BBa_B0030 in pSEVA251 plasmid.	Kan	This work
pSEVA251_ $P_{trc.x.lacO}$::*D-HicDH*	CDS of D-HicDH codon optimized for expression in *Synechocystis* sp. PCC 6803 under the control of the synthetic promoter $P_{trc.x.lacO}$ and the RBS BBa_B0030 in pSEVA251 plasmid.	Kan	This work

Amp: ampicillin; Kan: kanamycin.

Table S2. List of primers used in this study.

Primer name	Primer sequence 5'-3' *	Purpose	Reference /source
T7<br>T7term	TAATACGACTCACTATAGGG<br>TGCTAGTTATTGCTCAGCGG	pET21a(+) insert sequencing	Microsynth Austria
D-HicDH_BB_F<br>D-HicDH_BB_R	<u>GTTTCTTCGAATTCGCGGCCGCTTCTAG</u>ATGCATCATCACCACCACCATG<br><u>GTTTCTTCCTGCAGCGGCCGCTACTAGTA</u>TCATTATTTTGCTGGGCCGGTC	Amplification of CDS of D-HicDH codon optimized for expression in *Synechocystis* sp. PCC 6803 in the BioBrick format	This work
L-HicDH_BB_F<br>L-HicDH_BB_R	<u>GTTTCTTCGAATTCGCGGCCGCTTCTAG</u>ATGGCACGAAAAATTGGCATTATTG<br><u>GTTTCTTCCTGCAGCGGCCGCTACTAGTA</u>TTAAGCCGGATCTCAGTGGTGGTG	Amplification of CDS of L-HicDH codon optimized for expression in *Synechocystis* sp. PCC 6803 in the BioBrick format	This work
Ptrc.x.tetO2_F<br>Ptrc.x.tetO2_R	<u>AAAGAATTCGCGGCCGCTTCTAG</u>AGAGCTGTTGACAATTAATCA<br><u>AAACTGCAGCGGCCGCTACTAGTA</u>TTTCTCCTCTTTAATCTCTAGTATGTGTGAATCTCC	Amplification of $P_{trc.x.tetO2}$_B0030 in the BioBrick format	This work
Ptrc.x.lacO_F<br>Ptrc.x.lacO_R	<u>AAAGAATTCGCGGCCGCTTCTAG</u>AAATTGTGAGCGCTCACAATT<br><u>AAACTGCAGCGGCCGCTACTAGTA</u>TTTCTCCTCTTTAATCTCTAGTATGTGTGAAATTGTTA	Amplification of $P_{trc.x.lacO}$_B0030 in the BioBrick format	This work
PS1<br>PS2	AGGGCGGCGGATTTGTCC<br>GCGGCAACCGAGCGTTC	pSEVA251 insert amplification and sequencing	(Silva-Rocha et al., 2013)

*BioBrick^{TM} prefix (forward primer) and suffix (reverse primer) are underlined.

Table S3. List of *E. coli* strains used in this study.

Strain	Description	Purpose
E. coli NEB® 5-alpha	*fhuA2 (argF-lacZ)U169 phoA glnV44 80 (lacZ)M15 gyrA96 recA1 relA1 endA1 thi-1 hsdR17*	cloning
E. coli XL1-Blue	*recA1 endA1 gyrA96 thi-1 hsdR17 supE44 relA1 lac* [F′ *proAB* $lacI^q$*ZΔM15* Tn*10* (Tet^r)]	cloning
E. coli BL21(DE3)	*fhuA2 [lon] ompT gal (λ DE3) [dcm] ΔhsdS λ DE3 = λ sBamHIo ΔEcoRI-B int::(lacI::PlacUV5::T7 gene1) i21 Δnin5*	expression

Table S4. List of *Synechocystis* sp. PCC 6803 strains used in this study.

Strain	Description	References/sources
Synechocystis sp. PCC 6803	substrain Kazusa, geographical origin in California (USA)	Pasteur Culture Collection, Paris, France (Stanier et al., 1971; Trautmann et al., 2012; Kanesaki et al., 2012)
Synechocystis sp. PCC 6803 Δ*hoxYH*	*Synechocystis* sp. PCC 6803 mutant lacking the hydrogenase part of the Hox bidirectional hydrogenase	(Pinto et al., 2012)
Synechocystis sp. PCC 6803 Δ*hoxYH* pSEVA251_ P*trc.x.lacO::L-HicDH* (*S.* L-HicDH)	Transgenic *Synechocystis* sp. PCC 6803 constructed in Δ*hoxYH* background harbouring pSEVA251_$P_{trc.x.lacO}$*::L-HicDH.*	This work
Synechocystis sp. PCC 6803 Δ*hoxYH* pSEVA251_ P*trc.x.tetO2::D-HicDH* (*S.* D-HicDH)	Transgenic *Synechocystis* sp. PCC 6803 constructed in Δ*hoxYH* background harbouring pSEVA251_$P_{trc.x.tetO2}$*::D-HicDH.*	This work

Table S5. Specific activity of keto acid dehydrogenases tested as lyophilized cells in the presence of NAD(P)H, measured photometrically as decrease in NAD(P)H absorbance at 340 nm.

enzyme	substrate	cofactor	specific activity [U mg^{-1}]
L-2-Hydroxyisocaproic acid dehydrogenase from *Lactobacillus confuses* (L-HicDH)	**1a**	NADH	22 ± 1
		NADPH	4.7 ± 0.1
	**1b**	NADH	15 ± 5
		NADPH	3.6 ± 0.1
L-Lactate dehydrogenase from *Bacillus stearothermophilus* (L-LacDH)	**1a**	NADH	n.d.
		NADPH	n.d.
	**1b**	NADH	n.d.
		NADPH	n.d.
D-2-Hydroxyisocaproic acid dehydrogenase from *Lactobacillus paracasei* (D-HicDH)	**1a**	NADH	13 ± 8
		NADPH	13 ± 1
	**1b**	NADH	10 ± 1
		NADPH	4 ± 0
D-2-Hydroxy acid dehydrogenase from *Haloferax mediterranei* (D-HADH)	**1a**	NADH	n.d.
		NADPH	0.0028 ± 0.0001
	**1b**	NADH	n.d.
		NADPH	n.d.
YiaE from *E. coli* K12	**1a**	NADH	0.075 ± 0.018
		NADPH	0.45 ± 0.01
	**1b**	NADH	0.063 ± 0.018
		NADPH	0.20 ± 0.02

Reaction conditions: substrate (1.9 mM), NADPH (1.9 mM), potassium phosphate buffer (pH 7.0, 100 mM, 30 °C). L-HicDH 0.1 mg mL^{-1}, L-LacDH 0.3 mg mL^{-1}, D-HicDH 0.13 mg mL^{-1}, D-HADH 0.35 mg mL^{-1}, YiaE 0.1 mg mL^{-1}, lyophilized whole cells. Average and standard deviation of technical triplicates, background activity of reactions without substrate subtracted.

Table S6. Biotransformation of α-keto acids **1a-b** to α-hydroxy acids **2a-b**, catalysed by lyophilized whole *E. coli* cells expressing L- or D-HicDH, in the presence of NADPH.

enzyme	**1a**		**1b**	
	c. [%]	ee of **2a** [%]	c. [%]	ee of **2b** [%]
L-HicDH	86 ± 6	n.d.	90 ± 6	>99 (*S*)
D-HicDH	84 ± 5	n.d.	89 ± 22	>99 (*R*)

Reaction conditions: substrate 10 mM, NADPH 12 mM, potassium phosphate buffer (100 mM, pH 7.0), 30 °C, 120 rpm, 4 hours, enzymes in form of lyophilized whole cells; L-HicDH (pEG 220) 2 mg mL^{-1}, D-HicDH (pEG 221) 3.1 mg mL^{-1}. c., conversion; ee, enantiomeric excess; n.d., not determined.

Supporting Figures

Expression and activity of HicDHs in *E. coli* harbouring the genes codon optimized for *Synechocystis* sp. PCC 6803

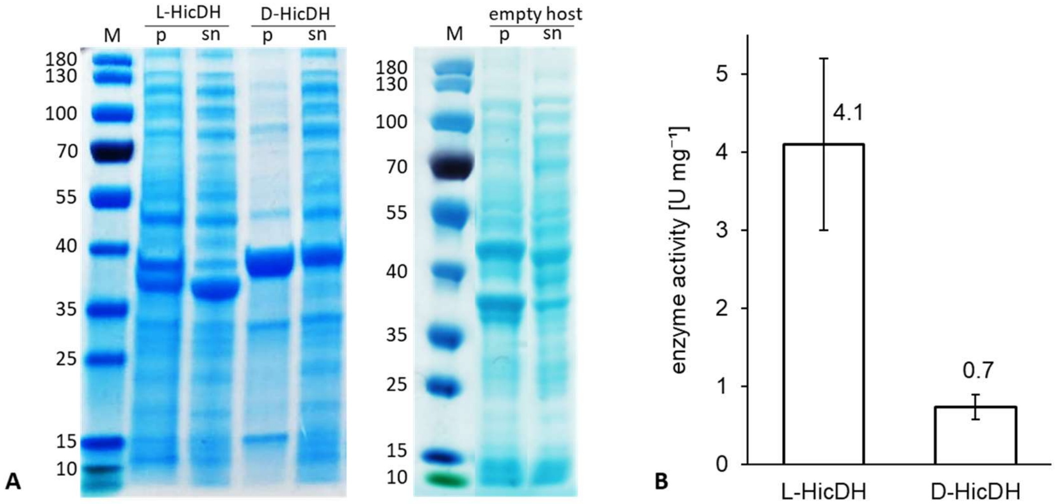

Figure S1. Expression of L-HicDH (34.2 kDa) and D-HicDH (37.9 kDa) from pET21a(+)_co_L-HicDH and pET21a(+)_co_D-HicDH (CDS codon optimized for *Synechocystis*) in *E. coli* BL21(DE3) cells. (**A**) SDS-PAGE gel. Cells were disrupted by sonication and 15 µg of protein loaded in each well; empty host: *E. coli* BL21(DE3); M, molecular weight maker; p, pellet; sn, supernatant. (**C**) Specific activity (units per mg of lyophilized whole cells) in the presence of NADPH with **1b**, measured photometrically as a decrease in NADPH absorbance at 340 nm. Reaction conditions: substrate (1.9 mM), NADPH (1.9 mM), potassium phosphate buffer (pH 7.5, 100 mM, 30 °C). L-HicDH 0.2 µg mL^{-1}, D-HicDH 0.4 µg mL^{-1} lyophilized cells. Average and standard deviation of technical triplicates.

Colony PCR of *S.* D-HicDH and *S.* L-HicDH

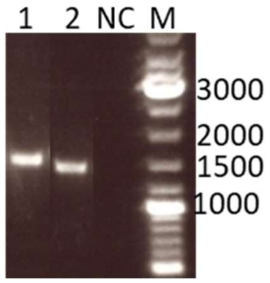

Figure S2. Colony PCR of *S.* D-HicDH and *S.* L-HicDH using primers PS1 and PS2. Product sizes for $P_{trc.x.lacO}$::*L-HicDH*: 1455 bp and $P_{trc.x.tetO2}$::*D-HicDH* 1421 bp. Lane 1: D-HicDH, Lane 2: *S.* L-HicDH, NC: no template control, M: molecular weight marker.

Growth curves of *Synechocystis* sp. PCC 6803 strains

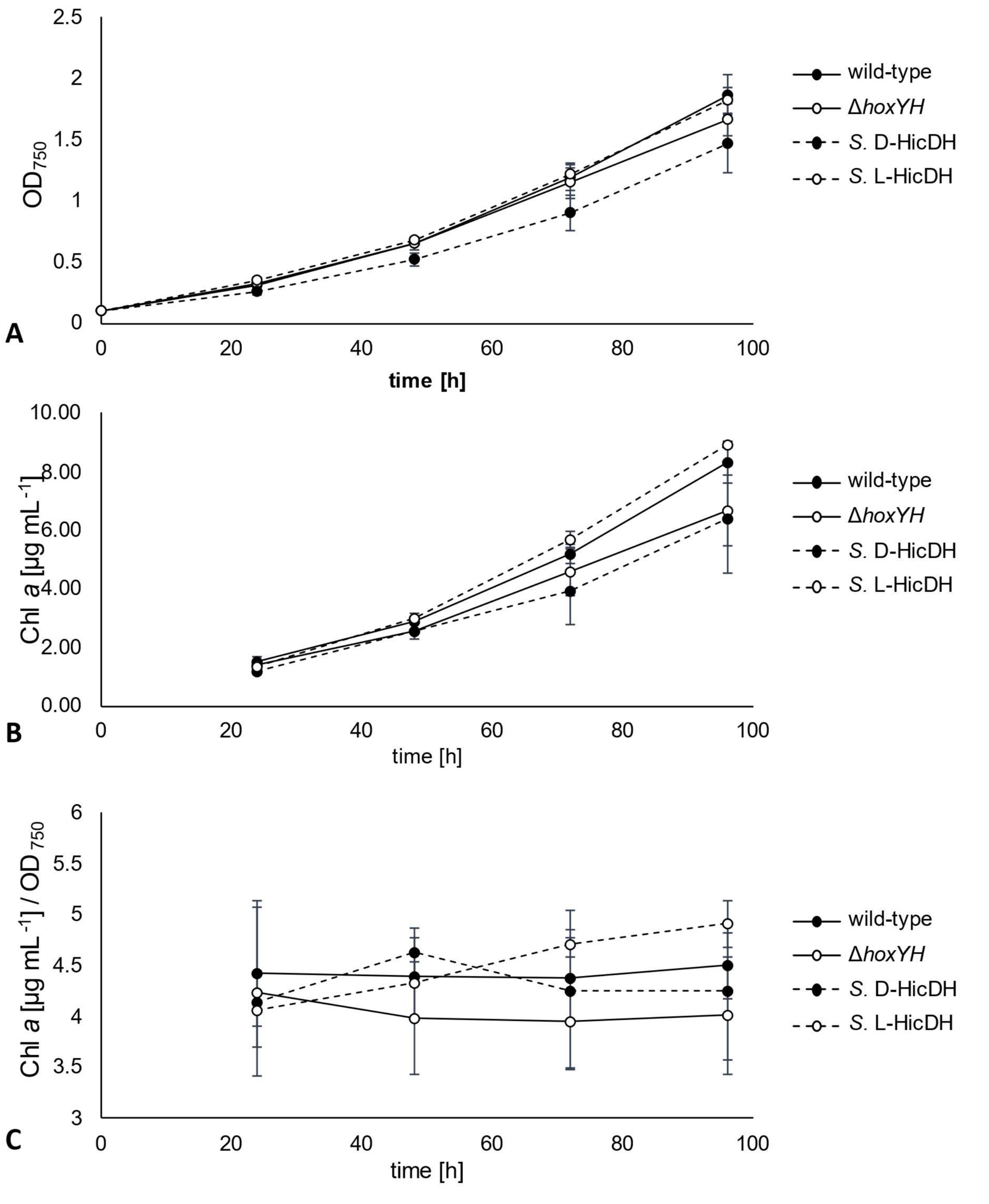

Figure S3. Growth curves of working cultures of *Synechocystis* sp. PCC 6803 strains showing (**A**) OD_{750}, (**B**) chlorophyll *a* content and (**C**) ratio of OD_{750} and chlorophyll *a* content depending on the time of cultivation. Cells were inoculated at OD_{750} 0.1 and grown under 80 µE m^{-2} s^{-1} light in 16 h light / 8 h dark at 30 °C while bubbling with sterile air.

In vivo product formation

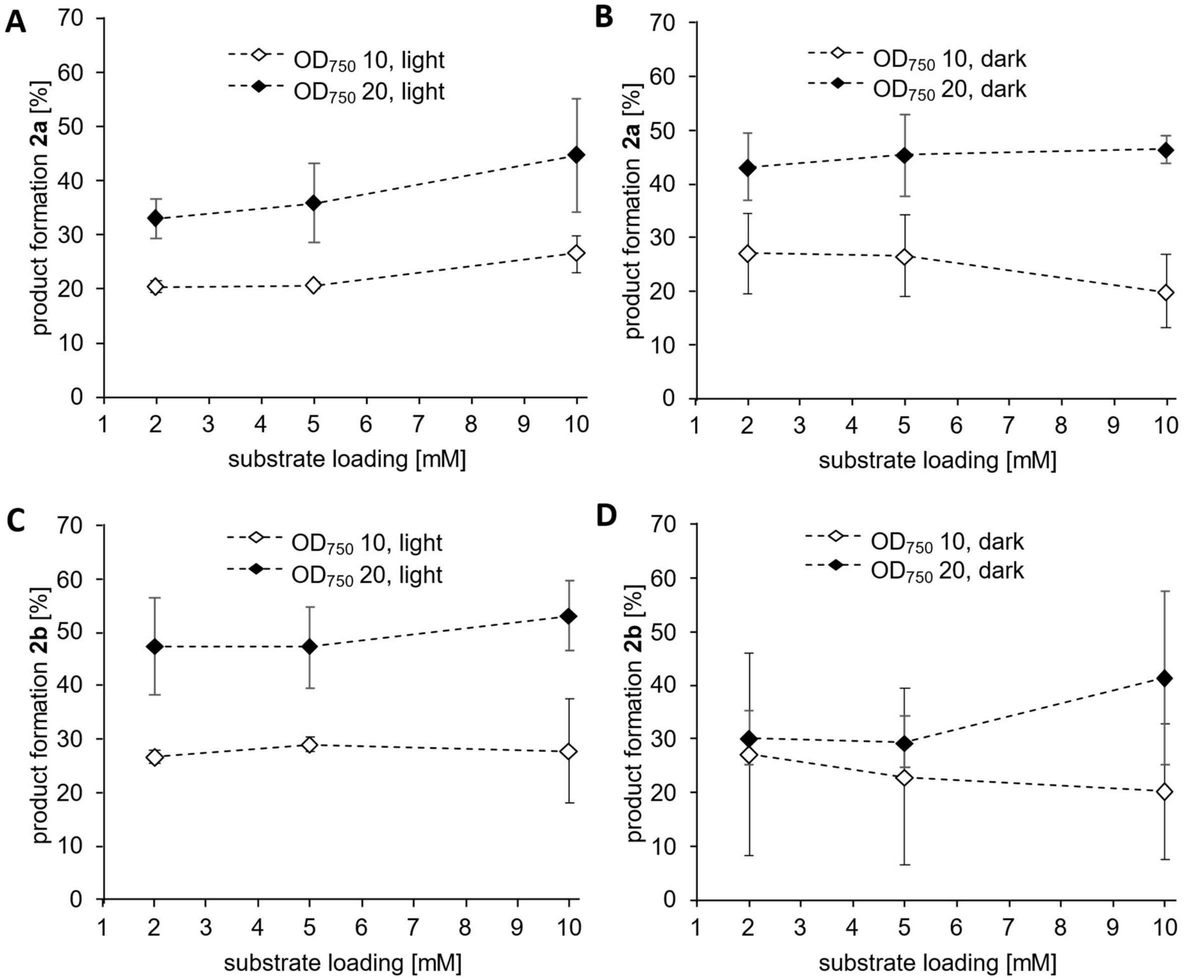

Figure S4. Dependence of product formation on substrate loading and cell density in *in vivo* biotransformations catalyzed by *S.* D-HicDH. (**A**) **2a**, light; (**B**) **2a**, dark; (**C**) **2b**, light, (**D**) **2b**, dark. Average and standard deviation of 3 independent experiments. Graph derived from the same data as shown in Figure 1.

In vivo control reactions

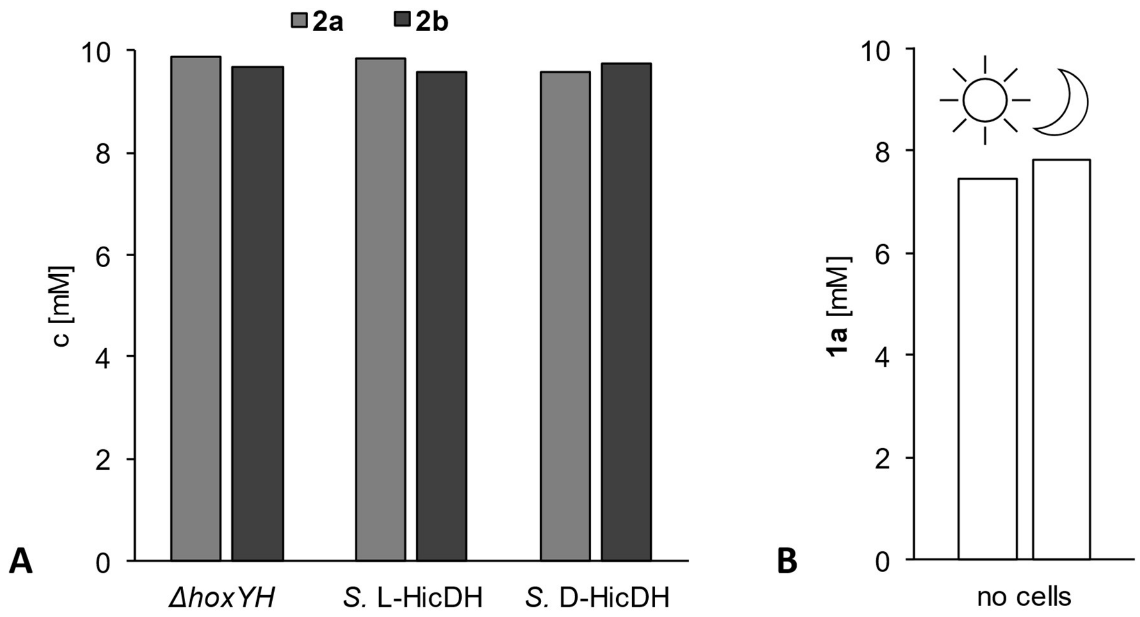

Figure S5. *In vivo* control reactions. (**A**) Recovery of *rac*-**2a** or *rac*-**2b** (10 mM) after incubation with cyanobacterial cells (OD_{750} = 10) under irradiation. (**B**) Recovery of **1a** after incubation in light or dark conditions, without cells. The reaction conditions correspond to *in vivo* biotransformations.

Representative chromatograms

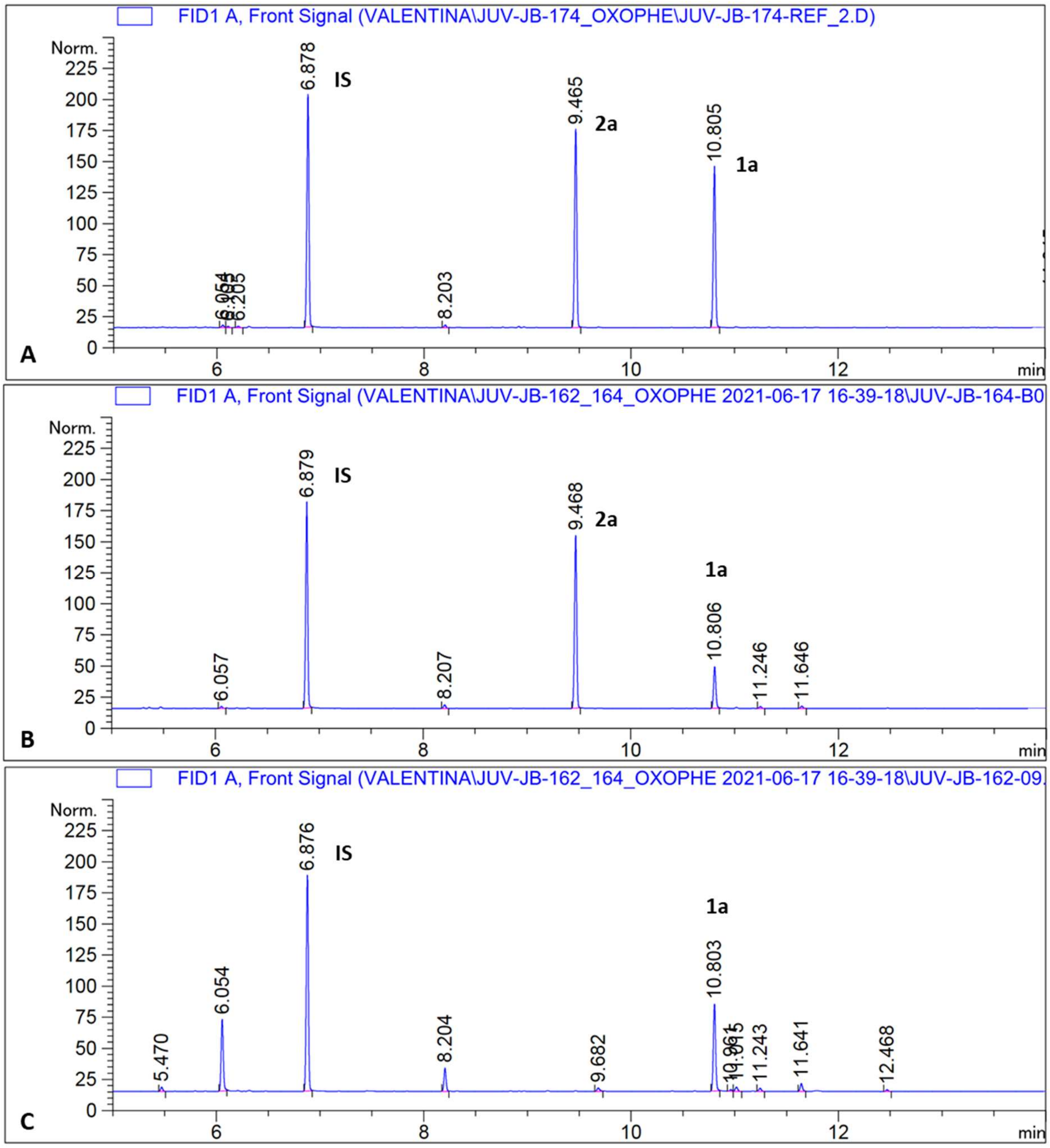

Figure S6. Examples of GC-FID chromatograms for quantification of **1a** and **2a** as trimethylsylil esters. (**A**) Commercially available references. (**B**) *In vivo* ketoreduction of **1a** (10 mM) by *Synechocystis* sp. PCC 6803 *ΔhoxYH* D-HicDH (OD_{750} = 20) in the presence of light. (**C**) *In vitro* reaction of **1a** (10 mM) with *Synechocystis* sp. PCC 6803 *ΔhoxYH cell* lysate and NADH (1 eq).

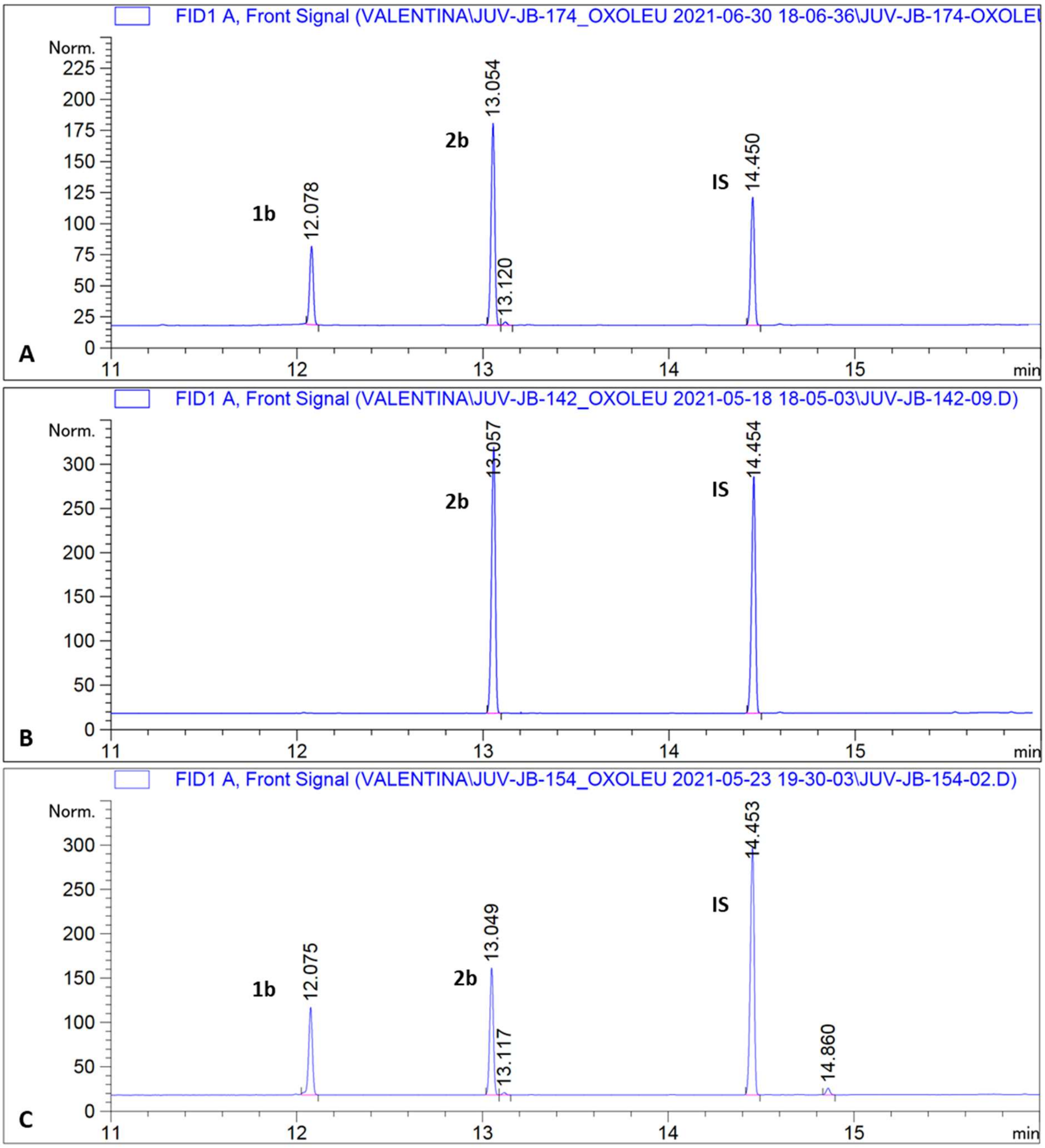

Figure S7. Examples of GC-FID chromatograms for quantification of **2a** and **2b** as trimethylsylil esters. (**A**) Commercially available references. (**B**) *In vitro* reaction of **2a** (10 mM) with *Synechocystis* sp. PCC 6803 *ΔhoxYH* D-HicDH cell lysate and NADH (1 eq). (**C**) *In vivo* ketoreduction of **2a** (10 mM) by *Synechocystis* sp. PCC 6803 *ΔhoxYH* D-HicDH (OD_{750} = 10) in the presence of light.

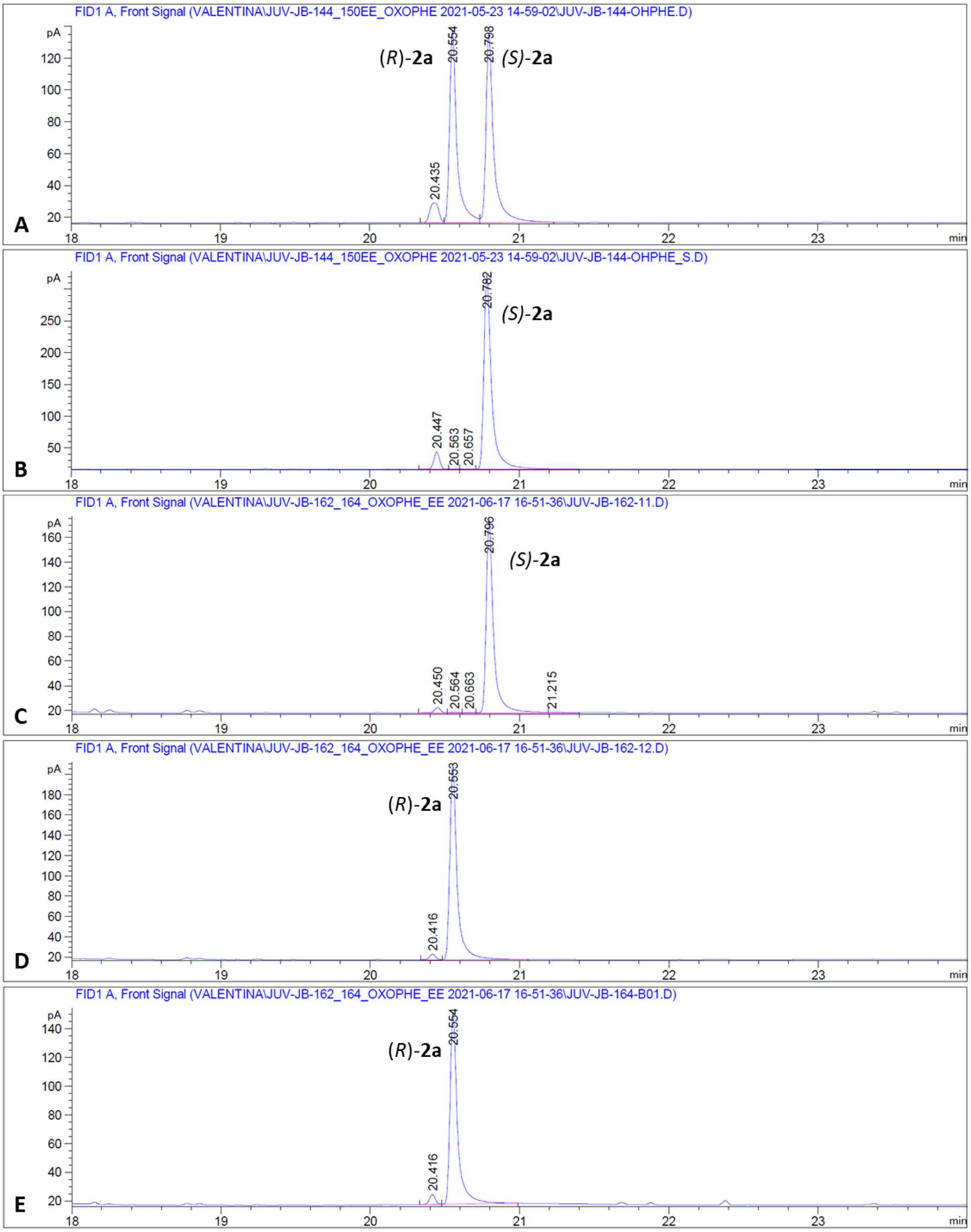

Figure S8. Examples of GC-FID chromatograms for determining the enantiomeric excess of **2a** methyl esters. (**A**) Commercially available reference of *rac*-**2a**. (**B**) Commercially available reference of (*S*)-**2b**. (**C**) *In vitro* ketoreduction of **1a** (10 mM) by *Synechocystis* sp. PCC 6803 *ΔhoxYH* L-HicDH cell lysate and NADH (1 eq). (**D**) *In vitro* ketoreduction of **1a** (10 mM) by *Synechocystis* sp. PCC 6803 *ΔhoxYH* D-HicDH cell lysate and NADH (1 eq). (**E**) *In vivo* ketoreduction of **1a** (10 mM) by *Synechocystis* sp. PCC 6803 *ΔhoxYH* D-HicDH (OD_{750} = 20) in the presence of light.

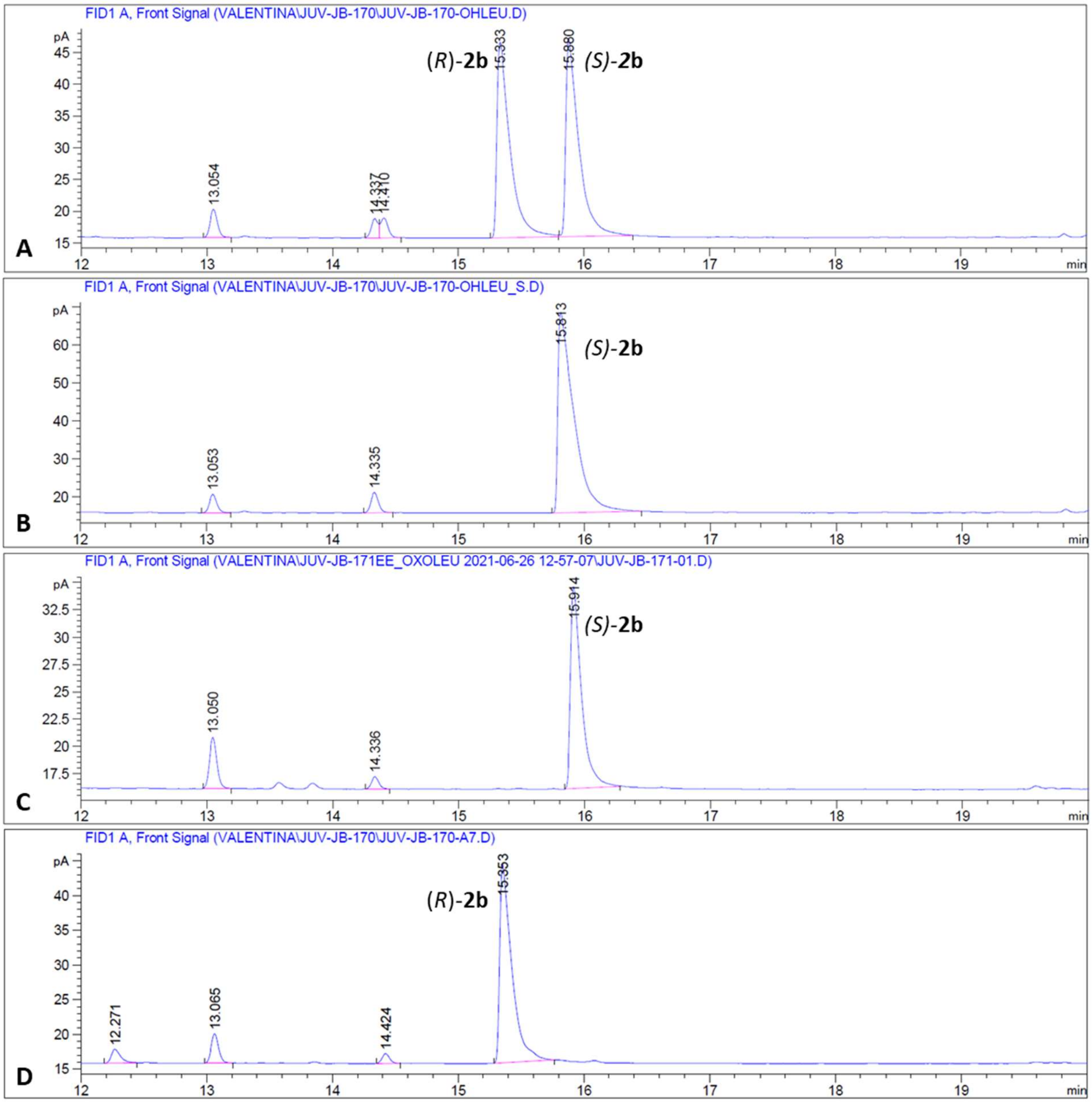

Figure S9. Examples of GC-FID chromatograms for determining the enantiomeric excess of **2b** methyl esters. (**A**) Commercially available reference of *rac*-**2b**. (**B**) Commercially available reference of (*S*)-**2b**. (**C**) *In vitro* ketoreduction of **1b** (10 mM) by *Synechocystis* sp. PCC 6803 *ΔhoxYH* L-HicDH cell lysate and NADH (1 eq). (**D**) *In vivo* ketoreduction of **1b** (10 mM) by *Synechocystis* sp. PCC 6803 *ΔhoxYH* D-HicDH (OD_{750} = 10) in the presence of light.

Calibration curves

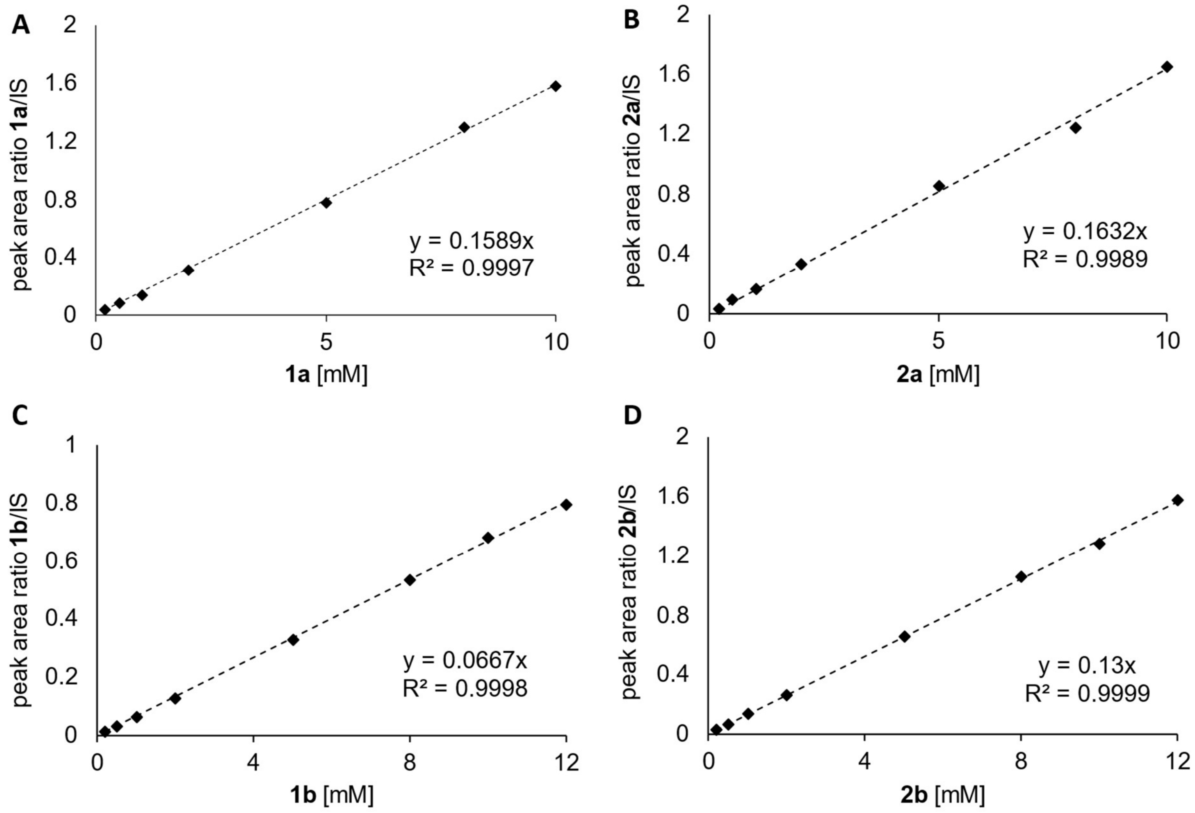

Figure S10. Calibration curves for the quantification of (**A**) **1a**, (**B**) **2a**, (**C**) **1b** and (**D**) **2b**. The corresponding line equations and R^2 values were generated by a simple linear regression and were forced through zero.

DNA and amino acid sequences

Sequences of D-HicDH and L-HicDH constructs in pSEVA251; CDS codon optimized for *Synechocystis* sp. PCC 6803; cloned under the control of $P_{trc.x.tetO2}$ and $P_{trc.x.lacO}$, respectively.

D-HicDH

plasmid: pSEVA251_ P*trc.x.tetO2::D-HicDH*

DNA sequence:

element	DNA sequence	AA sequence
BioBrick™ prefix[a]	GAATTCGCGGCCGCTTCTAGA	–
promoter $P_{trc.x.tetO2}$		–
scar1	TACTAGAG	–
RBS BBa.B0030		–
scar 2		–
start codon	**ATG**	–
N-terminal His-tag	CATCATCACCACCACCAT	HHHHHH
linker	GGTTCCGGT	GSG
stop	**TAATGA**	**
BioBrick™ suffix[b]	TACTAGTAGCGGCCGCTGCAG	–

[a]restriction sites EcoRI and XbaI underlined
[b]restriction sites SpeI and PstI underlined

GAATTCGCGGCCGCTTCTAGA
TACTAGAG **ATG**CATCATCACCACCA
CCATGGTTCCGGTAAGATTATTGCGTATGGAGCGCGGGTTGATGAGATCCAATACTTTAAGCAGTGGG
CTAAAGACACCGGGAATACCTTGGAGTATCACACCGAATTTCTTGATGAAAATACCGTTGAATGGGCC
AAGGGGTTCGACGGTATTAATAGTTTGCAAACAACCCCCTACGCGGCCGGCGTGTTTGAAAAAATGCA
CGCGTATGGGATCAAATTCTTAACCATTAGGAATGTGGGCACCGACAATATCGACATGACCGCCATGA
AACAGTATGGAATCCGCTTAAGTAACGTCCCCGCCTATAGTCCCGCCGCCATTGCCGAATTTGCATTG
ACAGACACCTTGTATCTGCTACGGAATATGGGGAAAGTGCAAGCCCAATTACAAGCGGGGGACTATGA
GAAAGCCGGCACCTTTATCGGAAAAGAACTAGGTCAACAGACCGTCGGCGTGATGGGTACCGGTCACA
TCGGCCAGGTGGCGATCAAATTGTTTAAAGGGTTTGGTGCTAAGGTTATAGCTTACGATCCGTATCCC
ATGAAAGGCGACCATCCCGATTTTGATTATGTGTCCCTTGAAGACCTGTTTAAACAAAGCGATGTCAT
CGACTTACACGTGCCTGGAATCGAACAGAACACGCATATCATCAATGAGGCCGCCTTTAATCTGATGA
AACCTGGTGCGATTGTGATTAACACCGCACGGCCTAACTTAATTGATACGCAGGCCATGCTCAGCAAC
TTGAAATCAGGCAAACTCGCAGGGGTGGGTATAGACACTTACGAATATGAAACTGAAGATCTTTTGAA
CCTGGCGAAGCACGGTTCCTTTAAAGATCCTTTGTGGGATGAATTATTAGGGATGCCGAATGTGGTAT
TGTCTCCTCATATCGCTTACTACACCGAAACTGCTGTGCATAATATGGTGTACTTTAGCCTTCAACAT
CTGGTGGATTTTCTGACTAAAGGCGAGACTTCTACAGAAGTGACCGGCCCAGCAAAA**TAATGA**TACTA
GTAGCGGCCGCTGCAG

Amino acid sequence:

MHHHHHHGSGKIIAYGARVDEIQYFKQWAKDTGNTLEYHTEFLDENTVEWAKGFDGINSLQTTPYAAG
VFEKMHAYGIKFLTIRNVGTDNIDMTAMKQYGIRLSNVPAYSPAAIAEFALTDTLYLLRNMGKVQAQL
QAGDYEKAGTFIGKELGQQTVGVMGTGHIGQVAIKLFKGFGAKVIAYDPYPMKGDHPDFDYVSLEDLF
KQSDVIDLHVPGIEQNTHIINEAAFNLMKPGAIVINTARPNLIDTQAMLSNLKSGKLAGVGIDTYEYE
TEDLLNLAKHGSFKDPLWDELLGMPNVVLSPHIAYYTETAVHNMVYFSLQHLVDFLTKGETSTEVTGP
AK**

L-HicDH

plasmid: pSEVA251_ P*trc.x.lacO::L-HicDH*

DNA sequence:

element	DNA sequence	AA sequence
BioBrick™ prefix[a]	GAATTCGCGGCCGCTTCTAGA	–
promoter $P_{trc.x.lacO}$		–
scar 1	TACTAGAG	–
RBS BBa.B0030		–
scar 2		–
start codon	**ATG**	–
XhoI site		LE
C-terminal His-tag	CACCACCACCACCACCAC	HHHHHH
stop	**TGA**	*
BioBrick™ suffix[b]	TACTAGTAGCGGCCGCTGCAG	–

[a]restriction sites EcoRI and XbaI underlined

[b]restriction sites SpeI and PstI underlined

```
GAATTCGCGGCCGCTTCTAGA
                                                       TACTAGAG
           ATGGCACGAAAAATTGGCATTATTGGCCTTGGCAACGTAGGCGCAGCCGTTGCCCA
CGGCTTAATTGCGCAAGGGGTTGCAGACGATTATGTGTTTATTGATGCGAACGAGGCCAAAGTGAAAG
CAGATCAGATTGATTTTCAGGATGCCATGGCGAATCTGGAAGCGCATGGGAATATTGTTATCAATGAT
TGGGCGGCTTTGGCTGATGCCGACGTTGTGATTTCCACTTTAGGAAACATTAAACTTCAGCAAGATAA
TCCCACCGGGGATAGATTTGCTGAATTAAAATTCACATCCTCCATGGTCCAGAGTGTGGGAACCAATC
TAAAAGAAAGTGGCTTTCATGGCGTGCTTGTGGTCATTTCCAATCCGGTGGATGTTATCACTGCACTT
TTTCAACACGTGACAGGCTTTCCCGCCCACAAGGTGATTGGTACCGGGACACTGCTCGATACTGCCAG
GATGCAAAGAGCTGTAGGTGAAGCCTTTGACCTGGATCCTCGCTCTGTTTCGGGCTACAACTTGGGGG
AGCACGGCAATAGCCAGTTTGTAGCCTGGAGTACTGTCCGGGTCATGGGGCAACCTATCGTAACCTTG
GCGGATGCCGGTGATATCGATCTGGCTGCTATTGAAGAAGAGGCTCGGAAAGGTGGCTTCACCGTCTT
AAACGGGAAGGGCTACACTTCGTATGGTGTAGCGACCAGCGCGATCCGAATTGCCAAAGCGGTGATGG
CCGACGCGCATGCCGAATTGGTGGTGAGTAATCGTCGTGACGATATGGGTATGTACCTGAGTTATCCG
GCCATTATCGGGCGTGACGGGGTGCTGGCTGAGACAACTTTAGACTTAACGACGGACGAACAAGAAAA
ACTGCTACAATCTAGGGACTACATTCAGCAGCGGTTTGATGAAATTGTTGACACTTTG      CACC
ACCACCACCACCACTGAGATCCGgctTAATACTAGTAGCGGCCGCTGCAG
```

Amino acid sequence:

```
MARKIGIIGLGNVGAAVAHGLIAQGVADDYVFIDANEAKVKADQIDFQDAMANLEAHGNIVINDWAAL
ADADVVISTLGNIKLQQDNPTGDRFAELKFTSSMVQSVGTNLKESGFHGVLVVISNPVDVITALFQHV
TGFPAHKVIGTGTLLDTARMQRAVGEAFDLDPRSVSGYNLGEHGNSQFVAWSTVRVMGQPIVTLADAG
DIDLAAIEEEARKGGFTVLNGKGYTSYGVATSAIRIAKAVMADAHAELVVSNRRDDMGMYLSYPAIIG
RDGVLAETTLDLTTDEQEKLLQSRDYIQQRFDEIVDTLLEHHHHHH*
```

References

Allen, M.M., 1968. Simple conditions for growth of unicellular blue-green algae on plates. J. Phycol. 4, 1–4. https://doi.org/10.1111/j.1529-8817.1968.tb04667.x

Bur, D., Luyten, M.A., Wynn, H., Provencher, L.R., Jones, J.B., Gold, M., Friesen, J.D., Clarke, A.R., Holbrook, J.J., 1989. An evaluation of the substrate specificity and asymmetric synthesis potential of the cloned L-lactate dehydrogenase from *Bacillus stearothermophilus*. Can. J. Chem. 67, 1065–1070. https://doi.org/10.1139/v89-161

Domenech, J., Ferrer, J., 2006. A new d-2-hydroxyacid dehydrogenase with dual coenzyme-specificity from *Haloferax mediterranei*, sequence analysis and heterologous overexpression. Biochim. Biophys. Acta BBA - Gen. Subj. 1760, 1667–1674. https://doi.org/10.1016/j.bbagen.2006.08.024

Englund, E., Liang, F., Lindberg, P., 2016. Evaluation of promoters and ribosome binding sites for biotechnological applications in the unicellular cyanobacterium *Synechocystis* sp. PCC 6803. Sci. Rep. 6. https://doi.org/10.1038/srep36640

Ferreira, E.A., Pacheco, C.C., Pinto, F., Pereira, J., Lamosa, P., Oliveira, P., Kirov, B., Jaramillo, A., Tamagnini, P., 2018. Expanding the toolbox for *Synechocystis* sp. PCC 6803: validation of replicative vectors and characterization of a novel set of promoters. Synth. Biol. 3. https://doi.org/10.1093/synbio/ysy014

Flores, H., Ellington, A.D., 2005. A modified consensus approach to mutagenesis inverts the cofactor specificity of *Bacillus stearothermophilus* lactate dehydrogenase. Protein Eng. Des. Sel. PEDS 18, 369–377. https://doi.org/10.1093/protein/gzi043

Gourinchas, G., Busto, E., Killinger, M., Richter, N., Wiltschi, B., Kroutil, W., 2015. A synthetic biology approach for the transformation of L-α-amino acids to the corresponding enantiopure (R)- or (S)-α-hydroxy acids. Chem. Commun. 51, 2828–2831. https://doi.org/10.1039/C4CC08286A

Holmberg, N., Ryde, U., Bülow, L., 1999. Redesign of the coenzyme specificity in l-Lactate dehydrogenase from *Bacillus stearothermophilus* using site-directed mutagenesis and media engineering. Protein Eng. Des. Sel. 12, 851–856. https://doi.org/10.1093/protein/12.10.851

Kanesaki, Y., Shiwa, Y., Tajima, N., Suzuki, M., Watanabe, S., Sato, N., Ikeuchi, M., Yoshikawa, H., 2012. Identification of Substrain-Specific Mutations by Massively Parallel Whole-Genome Resequencing of *Synechocystis* sp. PCC 6803. DNA Res. 19, 67–79. https://doi.org/10.1093/dnares/dsr042

Ludwig, A., Heimbucher, T., Gregor, W., Czerny, T., Schmetterer, G., 2008. Transformation and gene replacement in the facultatively chemoheterotrophic, unicellular cyanobacterium *Synechocystis* sp. PCC 6714 by electroporation. Appl. Microbiol. Biotechnol. 78, 729–735. https://doi.org/10.1007/s00253-008-1356-y

Meeks, J.C., Castenholz, R.W., 1971. Growth and photosynthesis in an extreme thermophile, *Synechococcus lividus* (Cyanophyta). Arch. Für Mikrobiol. 78, 25–41. https://doi.org/10.1007/BF00409086

Pinto, F., van Elburg, K.A., Pacheco, C.C., Lopo, M., Noirel, J., Montagud, A., Urchueguía, J.F., Wright, P.C., Tamagnini, P., 2012. Construction of a chassis for hydrogen production: physiological and

molecular characterization of a *Synechocystis* sp. PCC 6803 mutant lacking a functional bidirectional hydrogenase. Microbiology 158, 448–464. https://doi.org/10.1099/mic.0.052282-0

Silva-Rocha, R., Martínez-García, E., Calles, B., Chavarría, M., Arce-Rodríguez, A., de las Heras, A., Páez-Espino, A.D., Durante-Rodríguez, G., Kim, J., Nikel, P.I., Platero, R., de Lorenzo, V., 2013. The Standard European Vector Architecture (SEVA): a coherent platform for the analysis and deployment of complex prokaryotic phenotypes. Nucleic Acids Res. 41, D666–D675. https://doi.org/10.1093/nar/gks1119

Stanier, R.Y., Kunisawa, R., Mandel, M., Cohen-Bazire, G., 1971. Purification and properties of unicellular blue-green algae (order *Chroococcales*). Bacteriol. Rev. 35, 171–205. https://doi.org/10.1128/br.35.2.171-205.1971

Trautmann, D., Voß, B., Wilde, A., Al-Babili, S., Hess, W.R., 2012. Microevolution in Cyanobacteria: Re-sequencing a Motile Substrain of *Synechocystis* sp. PCC 6803. DNA Res. 19, 435–448. https://doi.org/10.1093/dnares/dss024

Villalobos, A., Ness, J.E., Gustafsson, C., Minshull, J., Govindarajan, S., 2006. Gene Designer: a synthetic biology tool for constructing artificial DNA segments. BMC Bioinformatics 7, 285. https://doi.org/10.1186/1471-2105-7-285

Yun, H., Choi, H.-L., Fadnavis, N.W., Kim, B.-G., 2005. Stereospecific Synthesis of (*R*)-2-Hydroxy Carboxylic Acids Using Recombinant *E. coli* BL21 Overexpressing YiaE from *Escherichia coli* K12 and Glucose Dehydrogenase from *Bacillus subtilis*. Biotechnol. Prog. 21, 366–371. https://doi.org/10.1021/bp049694w

Registry of Standard Biological Parts http://parts.igem.org/(accessed 4.1.21).
